# PI3Kδ Forms Distinct Multiprotein Complexes at the TCR Signalosome in Naïve and Differentiated CD4^+^ T Cells

**DOI:** 10.3389/fimmu.2021.631271

**Published:** 2021-03-08

**Authors:** Daisy H. Luff, Katarzyna Wojdyla, David Oxley, Tamara Chessa, Kevin Hudson, Phillip T. Hawkins, Len R. Stephens, Simon T. Barry, Klaus Okkenhaug

**Affiliations:** ^1^Laboratory of Lymphocyte Signalling and Development, The Babraham Institute, Cambridge, United Kingdom; ^2^Mass Spectrometry Facility, The Babraham Institute, Cambridge, United Kingdom; ^3^Signalling Programme, The Babraham Institute, Cambridge, United Kingdom; ^4^Bioscience, Oncology R&D, AstraZeneca, Cambridge, United Kingdom; ^5^Department of Pathology, University of Cambridge, Cambridge, United Kingdom

**Keywords:** PI3K, p110δ, TCR signalling, CD4^+^ T cells, interactomics, CRISPR-Cas9

## Abstract

Phosphoinositide 3-kinases (PI3Ks) play a central role in adaptive immunity by transducing signals from the T cell antigen receptor (TCR) via production of PIP_3_. PI3Kδ is a heterodimer composed of a p110δ catalytic subunit associated with a p85α or p85β regulatory subunit and is preferentially engaged by the TCR upon T cell activation. The molecular mechanisms leading to PI3Kδ recruitment and activation at the TCR signalosome remain unclear. In this study, we have used quantitative mass spectrometry, biochemical approaches and CRISPR-Cas9 gene editing to uncover the p110δ interactome in primary CD4^+^ T cells. Moreover, we have determined how the PI3Kδ interactome changes upon the differentiation of small naïve T cells into T cell blasts expanded in the presence of IL-2. Our interactomic analyses identified multiple constitutive and inducible PI3Kδ-interacting proteins, some of which were common to naïve and previously-activated T cells. Our data reveals that PI3Kδ rapidly interacts with as many as seven adaptor proteins upon TCR engagement, including the Gab-family proteins, GAB2 and GAB3, a CD5-CBL signalosome and the transmembrane proteins ICOS and TRIM. Our results also suggest that PI3Kδ pre-forms complexes with the adaptors SH3KBP1 and CRKL in resting cells that could facilitate the localization and activation of p110δ at the plasma membrane by forming ternary complexes during early TCR signalling. Furthermore, we identify interactions that were not previously known to occur in CD4^+^ T cells, involving BCAP, GAB3, IQGAP3 and JAML. We used CRISPR-Cas9-mediated gene knockout in primary T cells to confirm that BCAP is a positive regulator of PI3K-AKT signalling in CD4^+^ T cell blasts. Overall, our results provide evidence for a large protein network that regulates the recruitment and activation of PI3Kδ in T cells. Finally, this work shows how the PI3Kδ interactome is remodeled as CD4^+^ T cells differentiate from naïve T cells to activated T cell blasts. These activated T cells upregulate additional PI3Kδ adaptor proteins, including BCAP, GAB2, IQGAP3 and ICOS. This rewiring of TCR-PI3K signalling that occurs upon T cell differentiation may serve to reduce the threshold of activation and diversify the inputs for the PI3K pathway in effector T cells.

## Introduction

Class IA phosphoinositide 3-kinases (PI3Ks) play an essential role in cellular signal transduction by phosphorylating PI(4,5)P_2_ (PIP_2_) to generate the lipid second messenger PI(3,4,5)P_3_ (PIP_3_) (1). PI3K is a heterodimeric enzyme consisting of a p110 catalytic subunit, of which there are three class IA isoforms expressed in mammalian cells, p110α, p110β, and p110δ, associated with a p85 regulatory subunit, p85α/p55α/p50α, p85β, or p55γ (2). In contrast to p110α and p110β, which are ubiquitously expressed, p110δ is predominantly expressed in immune cells (2, 3).

T lymphocytes are key effectors of the adaptive immune response that are able to recognise and respond to antigens via the T cell antigen receptor (TCR). Activation of naïve CD4^+^ T cells, following engagement of the TCR and co-stimulatory receptors, is associated with dynamic changes in transcription and translation, leading to remodeling of the T cell proteome and differentiation into effector T helper (Th) cell populations (4).

These events are induced by a TCR signalling cascade that involves the rapid activation of PI3Kδ proximal to the receptor complex. p85α heterodimers are recruited to the immune synapse from the cytosol within a few seconds of antigen recognition, and the accumulation of PIP_3_ is observed rapidly at the plasma membrane following formation of the immune synapse (5–9). PI3Kδ is responsible for PIP_3_ production and AKT activation following TCR stimulation (9–11), and consequently plays a key role in antigen-induced CD4^+^ T cell proliferation, differentiation, and cytokine production (12–14).

Although the proximal and distal molecular events of the antigen-induced TCR signalling cascade have been intensely studied, the mechanism of PI3Kδ recruitment to the TCR signalosome remains unresolved (15). The recruitment and activation of p110δ depends upon high-affinity protein-protein interactions with the p85 subunit, which contains two SH2 domains that bind to phosphorylated tyrosine residues located within YxxM motifs (16). The p85 SH2:pYxxM interaction is important not only for localization of PI3Kδ to the plasma membrane, through binding to YxxM-containing adaptor proteins, but also for increasing PI3K activity via the release of inhibitory contacts between the p85 SH2 domains and the p110δ catalytic subunit (17, 18).

TCR signal transduction initially requires activation of the protein tyrosine kinases LCK and ZAP-70 and is followed by the assembly of scaffolding hubs nucleated by phosphorylated LAT and SLP-76, which allow for the activation of key downstream signalling pathways (19, 20). These proteins have also been proposed to recruit PI3K, despite lacking conventional YxxM motifs (21–24). Numerous other adaptors and YxxM-containing proteins are present at the TCR signalosome, and several have the capacity to bind to p85, but it is not clear which of these interactions are physiologically relevant as many were initially described in immortalised cell lines that may have rewired signalling networks (15, 25). Moreover, the proteins that connect the TCR to PI3K signalling have not been systematically analysed in an unbiased manner. Therefore, there is a need to identify the proteins that recruit PI3Kδ downstream of the TCR in primary T cells, and also to determine how they may change with the remodeling of the proteome during T cell differentiation.

To elucidate the mechanisms of PI3Kδ activation at the TCR signalosome, this study uses affinity purification coupled with quantitative mass spectrometry, biochemical approaches and CRISPR-Cas9 gene editing to uncover the p110δ interactome in both primary naïve CD4^+^ T cells and activated CD4^+^ T cell blasts. This work reveals a large protein network of cytosolic and transmembrane adaptors that connects PI3Kδ to the TCR and expands and rewires upon the differentiation of CD4^+^ T cells.

## Results

### Specific and Efficient Purification of p110δ Complexes From Primary CD4^+^ T Cells Using the AviTag-BirA System

**Figure 1:**
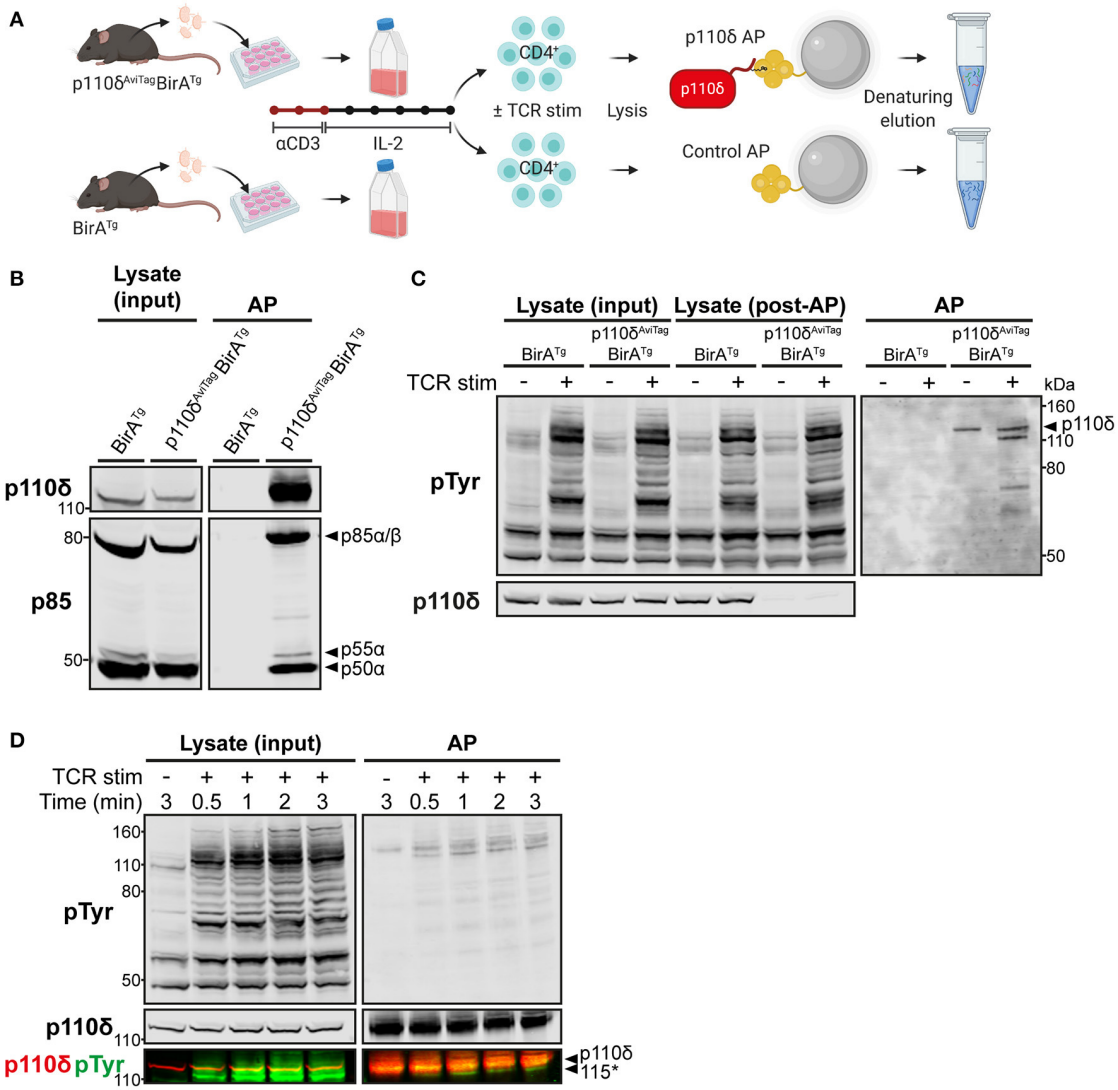
Specific affinity purification of p110δ complexes from primary CD4^+^ T cell blasts. **(A)** Schematic of the affinity purification (AP) protocol. T cells from the lymph nodes of p110δ^AviTag^BirA^Tg^ and BirA^Tg^ (control) mice were activated *in vitro* with anti-CD3 for 48 h and expanded for 5 days with IL-2. Purified CD4^+^ T cell blasts were then stimulated by CD3-CD4-crosslinking and their cell lysates were subjected to affinity purification (AP) using streptavidin-conjugated magnetic beads, as described in Materials and Methods. Proteins were eluted from the beads by denaturation then subjected to SDS-PAGE, before immunoblotting or nLC-MS/MS analysis. Schematic created with BioRender.com. **(B)** Immunoblot of control and p110δ APs from BirA^Tg^ and p110δ^AviTag^BirA^Tg^ CD4^+^ T cell blasts, respectively, alongside the whole cell lysate inputs. The membrane was probed with anti-p110δ and anti-pan-p85, which detects all isoforms of p85. Immunoblot from one experiment representative of at least three independent experiments. **(C)** Immunoblot of control and p110δ APs from BirA^Tg^ and p110δ^AviTag^BirA^Tg^ CD4^+^ T cell blasts, respectively, that had been stimulated for 1 min by CD3-CD4-crosslinking (TCR stim; +) or were left unstimulated (–), alongside whole cell lysates before (input) and after (post-AP) affinity purification to show efficient depletion of p110δ. Membranes were probed with anti-phosphotyrosine (pTyr), to detect phosphorylated tyrosine residues, and anti-p110δ. The black arrowhead indicates p110δ (119.7 kDa). Immunoblot from one experiment representative of at least three independent experiments. **(D)** Immunoblot of p110δ APs and whole cell lysates from p110δ^AviTag^BirA^Tg^ CD4^+^ T cell blasts that had been stimulated for the indicated times by CD3-CD4-crosslinking (TCR stim; +). Bands detected by anti-p110δ and anti-pTyr are overlaid in the bottom panel to show that the unknown co-purified pTyr-protein of ~115 kDa (labelled 115*) runs below p110δ (119.7 kDa). Immunoblot from one experiment representative of two independent experiments.

To determine the components of the p110δ interactome in CD4^+^ T cells required a specific and efficient method to isolate endogenous p110δ protein complexes from primary T cells. We therefore made use of the newly developed p110δ-AviTag transgenic mouse, *Pik3cd*^Avi/Avi^, which expresses p110δ protein with a C-terminal AviTag from the endogenous *Pik3cd* locus (26). p110δ kinase activity was found to be functional, albeit slightly impaired, in lymphocytes from *Pik3cd*^Avi/Avi^ mice (Supplementary Figure 1). These mice were crossed with *Rosa26*^BirA/BirA^ mice that express the biotin ligase BirA to generate *Pik3cd*^Avi/Avi^*Rosa26*^BirA/+^ progeny (hereafter called p110δ^AviTag^BirA^Tg^). In these mice, p110δ is constitutively biotinylated *in vivo* by BirA at the AviTag sequence and can therefore be rapidly affinity purified from cell lysates using streptavidin-conjugated magnetic beads (Figure 1A).

To validate the use of this system, T cells from the lymph nodes of p110δ^AviTag^BirA^Tg^ mice were activated for 2 days with anti-CD3 and then expanded for 5 days with interleukin-2 to generate differentiated T cell blasts. Lysates from purified CD4^+^ T cell blasts were subjected to streptavidin-mediated affinity purification (AP) to isolate p110δ complexes. Control purifications were also carried out using CD4^+^ T cell blasts from BirA^Tg^ mice, expressing wild-type p110δ, in order to account for background proteins that associate non-specifically with the beads or are otherwise biotinylated and therefore bind to streptavidin. Immunoblots of the bead eluates confirmed that p110δ could be specifically and efficiently purified from p110δ^AviTag^BirA^Tg^ CD4^+^ T cells, and not from BirA^Tg^ control cells (Figures 1B,C). The p85 regulatory subunits, p85α, p55α and p50α, were also successfully co-purified specifically with p110δ, at ratios comparable to those of their observed expression levels in cell lysates (Figure 1B).

To determine whether TCR signalling-induced p110δ complexes could be isolated using this approach, affinity purifications were carried out from CD4^+^ T cell blasts that had been stimulated *in vitro* by anti-CD3 plus anti-CD4 crosslinking for 1 min. This stimulation method mimics TCR-CD4 engagement by peptide-MHC and enables recruitment of CD4-associated LCK to the receptor complex for maximal tyrosine phosphorylation of proteins in the TCR signalling cascade [(27); Supplementary Figure 2]. Strikingly, several tyrosine-phosphorylated proteins were co-purified specifically with p110δ from TCR-stimulated cells, and not from unstimulated nor BirA^Tg^ control cells (Figure 1C). In particular, a prominently tyrosine-phosphorylated protein of ~115 kDa associated with p110δ rapidly upon TCR stimulation (Figures 1C,D). This tyrosine-phosphorylated protein migrated faster than the ~120 kDa p110δ protein band by SDS-PAGE, indicating that it was not a phosphorylated form of p110δ.

These results demonstrated that the AviTag-mediated affinity purification approach enabled rapid and selective isolation of endogenous p110δ and associated phospho-proteins from primary CD4^+^ T cell blasts.

### Identification of the p110δ Interactome in CD4^+^ T Cell Blasts by AP-MS

**Figure 2:**
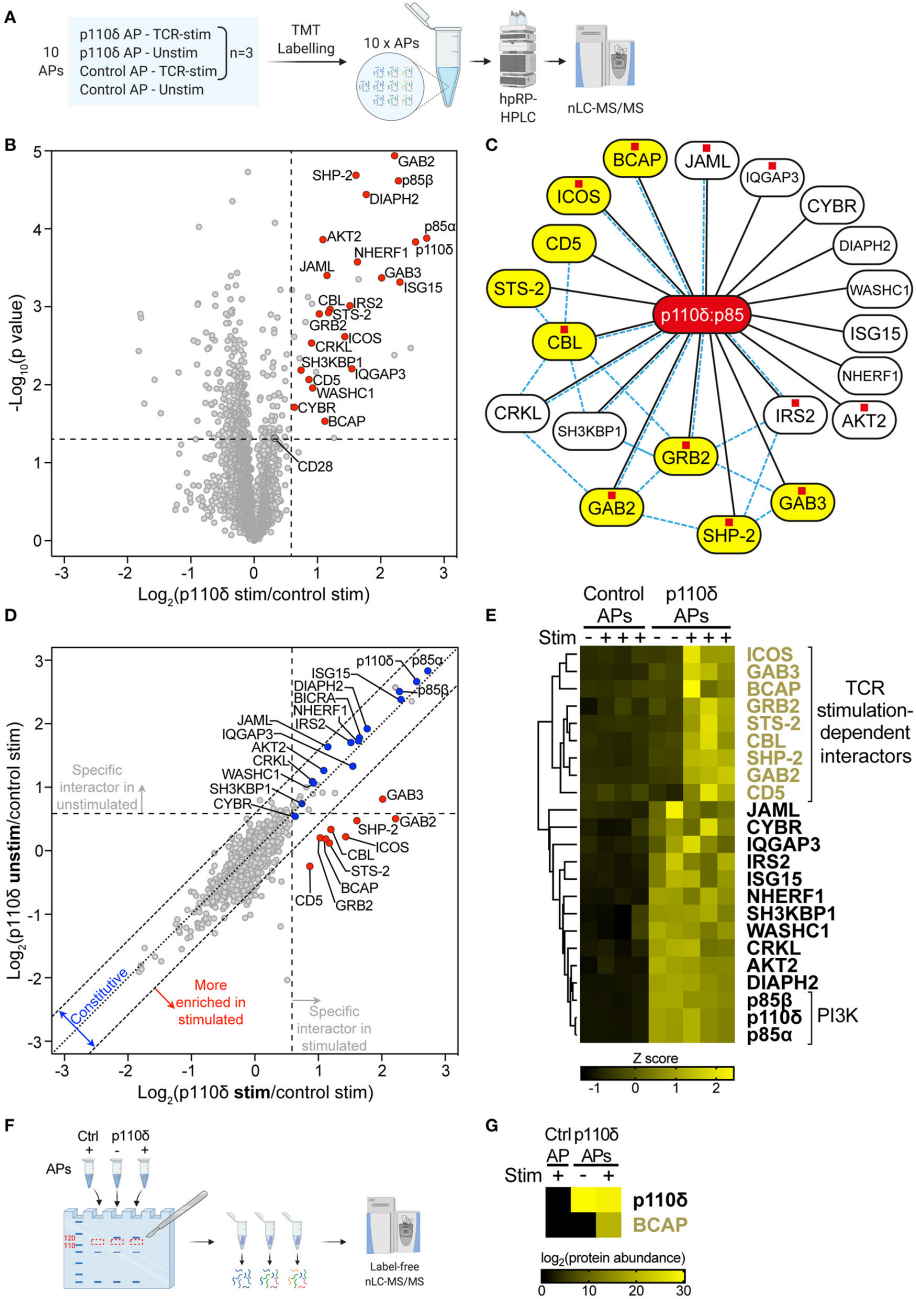
The p110δ interactome in CD4^+^ T cell blasts. **(A)** Schematic of samples analysed by quantitative MS. p110δ and control APs were produced from unstimulated and TCR-stimulated CD4^+^ T cell blasts in three independent biological-repeat experiments (detailed in Supplementary Figures 3A,B). Peptides were generated from the AP bead eluates then labelled in parallel with 10plex™ isobaric Tandem Mass Tags (TMTs) and combined for high-pH reversed-phase HPLC (hpRP-HPLC) pre-fractionation followed by nLC-MS/MS. **(B)** Volcano plot of proteins identified by mass spectrometry in APs from CD4^+^ T cell blasts that had been stimulated for 1 min by CD3-CD4-crosslinking. The plot shows the log_2_-difference in abundance of each protein in p110δ APs compared to control APs from three independent repeat experiments [Log_2_(p110δ stim/control stim)] vs. the –Log_10_-*p* value, determined by a two-tailed Student's *t*-test. The thresholds used to determine specific p110δ-interactors (upper-right quadrant) are drawn at 1.5-fold enrichment and *p* = 0.05. Proteins of interest are represented by red points. CD28 was classed a non-specific background protein. The full list of all proteins identified and *t*-test results can be found in Supplementary Datasheet 1. **(C)** Network diagram illustrating the specific p110δ-interactors identified by AP-MS in TCR-stimulated CD4^+^ T cell blasts (connected to p110δ with black lines). Previously known direct protein-protein interactions with p85 or between p110δ-interactors are indicated with pale blue dashed lines, as referenced in Supplementary Table 1. Proteins containing at least one YxxM motif are labelled with a red square, as listed in Supplementary Table 2. TCR stimulation-dependent p110δ-interactors are coloured yellow. **(D)** Scatter plot comparing the abundance of identified proteins in p110δ purifications from TCR-stimulated and unstimulated T cell blasts, relative to their abundance in control APs, from three independent repeat experiments. The diagonal dotted line indicates no difference between stimulated and unstimulated cells, while the diagonal dashed lines represent thresholds of 1.5-fold enrichment in either condition. The thresholds used to determine specific p110δ-interactors are drawn as vertical and horizontal dashed lines for stimulated and unstimulated cells, respectively, indicating 1.5-fold enrichment in p110δ APs compared to control APs. Specific interactors of interest are coloured blue (constitutive) and red (stimulation-induced). **(E)** Heatmap visualizing the Z score-normalised log_2_-protein abundance of specific p110δ-interactors in control and p110δ APs from unstimulated (–) and TCR-stimulated (+) CD4^+^ T cell blasts from three independent experiments. The result of hierarchical clustering of the data is represented by a dendrogram. **(F)** Control and p110δ APs from unstimulated (–) and TCR-stimulated (+) CD4^+^ T cell blasts were separated by SDS-PAGE. For each sample lane, the region corresponding to 110–120 kDa was excised from the gel and peptides were extracted, digested and analysed by label-free nLC-MS/MS. **(G)** Heatmap visualizing the log_2_-protein abundance of p110δ and BCAP in the 110–120 kDa gel slice of the three APs as described in **(F)**. Black cells indicate that the protein was not identified in that sample. The full list of all proteins identified can be found in Supplementary Datasheet 2.

The proteins co-purified with p110δ from CD4^+^ T cell blasts were next identified in an unbiased manner using quantitative mass spectrometry. For this, p110δ and control affinity purifications were carried out from the lysates of unstimulated and TCR-stimulated CD4^+^ T cell blasts and peptides were generated from the purified proteins by tryptic digestion. Peptide samples from three independent experiments were labelled with isobaric Tandem Mass Tags [TMTs; (28)] and combined for high-pH reversed-phase pre-fractionation followed by nano-liquid chromatography-tandem mass spectrometry (nLC-MS/MS) to enable relative quantification of the abundance of each identified protein in each of the AP samples (Figure 2A; Supplementary Figures 3A,B).

To distinguish p110δ-interactors from irrelevant background proteins, the abundance of each protein identified by MS was compared between p110δ APs and control APs from TCR-stimulated cells (Figure 2B; Supplementary Datasheet 1). Specific p110δ-interactors were defined as those proteins enriched >1.5-fold (*p* < 0.05) in p110δ purifications compared to control purifications. The validity of this approach was confirmed by the identification of the PI3K regulatory subunits, p85α and p85β, as highly-enriched specific p110δ-interactors. Strikingly, nine of the identified p110δ-interactors had previously been shown to have the capacity to bind directly to p85 (Figure 2C; Supplementary Table 1). Furthermore, eleven of the p110δ-interactors contain at least one YxxM motif within their amino acid sequence (Figure 2C; Supplementary Table 2). Taken together, this gives confidence that the proteins identified by AP-MS are likely to be true p110δ-interactors, and also suggests that many are direct, rather than indirect, binding partners of the p110δ:p85 heterodimer.

Fourteen of the identified p110δ-interactors are known to be involved in the regulation of TCR signalling, including the co-receptors ICOS and CD5, the adaptor proteins GRB2, GAB2, GAB3, CRKL, SH3KBP1, CYBR and NHERF1, the protein phosphatases STS-2 and SHP-2, the E3 ligase and adaptor CBL, the effector kinase AKT2, and the WASH complex component WASHC1. Of these proteins, ICOS, GRB2, GAB2, CRKL, SH3KBP1 and CBL have previously been shown to be able to interact directly with p85 (Figure 2C; Supplementary Table 1). Notably, nine of the TCR-signalling proteins are able to bind directly to at least one of the other identified p110δ-interactors (Figure 2C; Supplementary Table 1). This suggests that p110δ may be recruited to several multivalent protein complexes at the TCR signalosome in CD4^+^ T cell blasts.

The remaining proteins identified as specific p110δ-interactors have not previously been shown to play a role in TCR signal transduction: the adaptor proteins BCAP, IRS2 and IQGAP3, the co-receptor JAML, and the formin DIAPH2. However, BCAP, IRS2 and JAML can bind directly to p85 (29, 30). Most intriguingly, BCAP functions as a key adaptor for PI3K in B lymphocytes by recruiting p85 to the B-cell antigen receptor (BCR) upon antigen engagement (29, 31). Therefore, the identification of BCAP in the p110δ interactome suggested that it could perform a similar role downstream of the TCR in CD4^+^ T cell blasts.

To determine whether BCAP and the other p110δ-interactors could be involved in antigen-induced recruitment of p110δ to the TCR signalosome, the stimulation-dependency of their interactions with p110δ were examined. For this, the abundance of each p110δ-interactor was compared between p110δ APs from unstimulated and TCR-stimulated blasts, with respect to its abundance in control APs, from the three independent experiments (Figure 2D). Hierarchical clustering was also performed to group proteins that associated with p110δ similarly across all samples (Figure 2E). Strikingly, a distinct group of nine proteins was more highly enriched in p110δ purifications from TCR-stimulated cells than in those from unstimulated cells. ICOS, GAB3, BCAP, GRB2, STS-2, CBL, SHP-2, GAB2 and CD5 associated with p110δ in a TCR stimulation-dependent manner, with low abundance in unstimulated p110δ purifications that was comparable to their abundance in control purifications. These proteins are therefore most likely to be involved in the recruitment and activation of p110δ at the TCR signalosome.

The remaining proteins did not demonstrate stimulation-dependency in their association with p110δ, and therefore are likely to be constitutively associated with p110δ in both unstimulated and stimulated cells. As expected, p85α and p85β were both found to be constitutively associated with p110δ. Intriguingly, among the other constitutive p110δ-interactors, CRKL, SH3KBP1 and IRS2 are able to bind directly to several of the proteins that were found to associate with p110δ in a TCR-stimulation dependent manner (Figure 2C; Supplementary Table 1). Thus, these results suggest that p110δ forms complexes in resting, unstimulated cells that subsequently facilitate its recruitment to ternary complexes at the TCR signalosome upon stimulation.

It was plausible that one of the nine TCR stimulation-dependent p110δ-interactors corresponded with the prominently tyrosine-phosphorylated protein of ~115 kDa observed in immunoblots of p110δ APs from stimulated cells. To investigate this further, an additional set of control and p110δ APs from unstimulated and TCR-stimulated CD4^+^ T cell blasts were separated by SDS-PAGE and proteins that migrated within the gel band corresponding to 110–120 kDa were subjected to nLC-MS/MS for identification and label-free quantification (Figure 2F; Supplementary Figure 3C). Intriguingly, BCAP was identified in the p110δ AP from TCR-stimulated cells, but not in the p110δ AP from unstimulated cells nor in the control AP (Figure 2G; Supplementary Datasheet 2). This suggests that BCAP is a TCR-induced specific PI3Kδ-interactor, and that BCAP is rapidly tyrosine-phosphorylated following TCR engagement in CD4^+^ T cell blasts.

### BCAP Is Upregulated in Activated CD4^+^ T Cells and Tyrosine-Phosphorylated Downstream of the TCR

**Figure 3:**
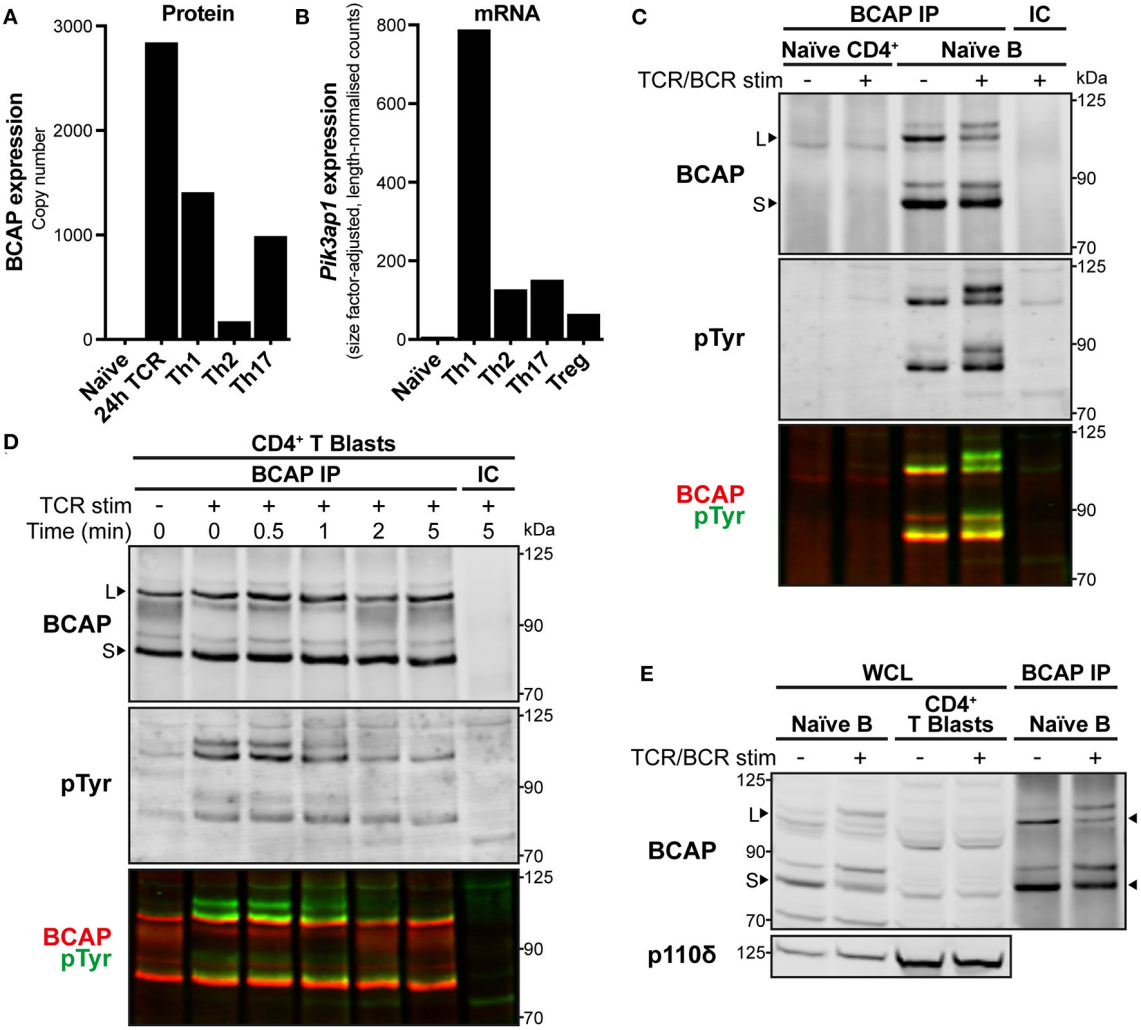
BCAP is upregulated in activated CD4^+^ T cells and tyrosine-phosphorylated upon TCR stimulation. **(A)** BCAP protein copy number in mouse naïve CD4^+^ T cells, 24 h-antigen-activated OT-II CD4^+^ T cells, and differentiated Th1, Th2, and Th17 CD4^+^ T cells. Data are from ImmPRes [http://immpres.co.uk; (4)]. **(B)** Expression of *Pik3ap1* mRNA in murine naïve splenic CD4^+^ T cells, *in vitro*-activated and polarised Th1, Th2, and Th17 CD4^+^ T cells, and splenic Tregs. Data are from Th-Express [https://th-express.org; (33)]. Expression levels in each subtype are calculated as read counts normalised by size factor and transcript length. **(C)** Immunoblot of immunoprecipitates (IPs) from naïve CD4^+^ T cells and naïve splenic B cells using anti-BCAP (BCAP IP) or IgG isotype-control antibody (IC). Naïve CD4^+^ T cells were stimulated by CD3-CD4-crosslinking for 30 s and naïve B cells were stimulated with anti-mouse IgM F(ab′ )_2_ for 2.5 min (TCR/BCR stim; +), or left unstimulated (–). The membrane was probed with anti-BCAP and anti-pTyr and the signals detected for each are overlaid in the third panel in red and green, respectively. Arrowheads indicate the BCAP long (L) and short (S) isoforms. Immunoblot representative of two independent experiments. **(D)** Immunoblot of IPs from CD4^+^ T cell blasts using anti-BCAP (BCAP IP) or IgG isotype-control antibody (IC). Cells were stimulated by CD3-CD4-crosslinking (TCR stim; +) for the indicated times or left unstimulated (–). The membrane was probed with anti-BCAP and anti-pTyr and the signals detected for each are overlaid in the third panel in red and green, respectively. Arrowheads indicate the BCAP long (L) and short (S) isoforms. Immunoblot from one experiment representative of three independent experiments. **(E)** Immunoblot of whole cell lysates (WCL) from an equal number of naïve splenic B cells and CD4^+^ T cell blasts, alongside BCAP IPs from naïve B cells to show the location of BCAP long (L) and short (S) isoforms, indicated with arrowheads. Naïve B cells were stimulated with anti-mouse IgM F(ab′ )_2_ for 2.5 min and CD4^+^ T cell blasts were stimulated by CD3-CD4-crosslinking for 1 min (TCR/BCR stim; +), or left unstimulated (–). The membrane was probed with anti-BCAP and anti-p110δ as a loading reference. Immunoblot representative of at least three independent experiments.

It was initially surprising to identify BCAP in the p110δ interactome as previous studies that focused on the role of BCAP in B cells did not detect BCAP protein in naïve T cells (32). Therefore, we examined the levels of BCAP protein in naïve CD4^+^ T cells as well as antigen-activated and effector CD4^+^ T cells using the open-access Immunological Proteomic Resource [ImmPRes; (4)]. BCAP was not detected in naïve CD4^+^ T cells but was upregulated within 24 h of antigen activation and sustained upon *in vitro* differentiation into Th1 effector CD4^+^ T cells (Figure 3A). To determine if BCAP levels in T cells were regulated at the transcriptional level, expression of the gene encoding BCAP (*Pik3ap1*) was investigated using mRNA sequencing data from the publicly-available murine CD4^+^ T cell transcriptome atlas (33). Strikingly, *Pik3ap1* expression was extremely low in naïve CD4^+^ cells, but was greatly upregulated in *in vitro*-differentiated Th1 CD4^+^ T cells (Figure 3B).

The expression of BCAP protein in CD4^+^ T cells was then examined further by immunoprecipitation with anti-BCAP polyclonal antibodies. *Pik3ap1* is expressed in mouse B cells as two protein isoforms [Figure 3C; long (L) and short (S); (29)]. In accordance with the lack of expression in naïve CD4^+^ T cells, neither BCAP protein isoform could be isolated from naïve CD4^+^ T cells (Figure 3C). Conversely, both long and short isoforms of BCAP were detected in CD4^+^ T cell blasts following immunoprecipitation (Figure 3D). BCAP could not be distinguished in whole cell lysates from CD4^+^ T cell blasts by immunoblotting with anti-BCAP (Figure 3E), indicating that BCAP protein was present at lower levels in CD4^+^ T cell blasts compared to naïve B cells, as well as demonstrating the low sensitivity of the anti-BCAP antibodies.

Earlier MS analyses suggested that BCAP could be the tyrosine-phosphorylated ~115 kDa protein present in p110δ APs from TCR-stimulated cells. Indeed, mouse BCAP contains 23 tyrosine residues, including four located within YxxM motifs (Supplementary Table 2), and is tyrosine-phosphorylated in naïve B cells [Figure 3C; (29)]. The BCAP YxxM motifs are required for its association with p85 in chicken DT40 B cells upon BCR stimulation (29). To investigate whether BCAP is also tyrosine-phosphorylated in CD4^+^ T cell blasts, BCAP was immunoprecipitated from unstimulated and TCR-stimulated cells and the IPs were immunoblotted for phosphotyrosine residues. BCAP was rapidly tyrosine-phosphorylated upon CD3-CD4-crosslinking in CD4^+^ T cell blasts (Figure 3D). Furthermore, p85 could be detected in BCAP immunoprecipitates equally rapidly upon TCR crosslinking, indicating that TCR signalling induces BCAP tyrosine-phosphorylation and association with PI3K (Supplementary Figure 4).

Collectively, these results demonstrate that BCAP is expressed in CD4^+^ T cells following their activation and is regulated by phosphorylation during early TCR signalling. Combined with the earlier identification of BCAP as a TCR stimulation-dependent p110δ-interaction partner, these observations suggested that BCAP may be involved in the recruitment and activation of p110δ at the TCR signalosome in CD4^+^ T cell blasts, analogous to its role in BCR signalling.

### BCAP Positively Regulates p110δ-AKT Signalling in Activated CD4^+^ T Cells

To investigate a potential role for BCAP in the regulation of p110δ activity downstream of the TCR, PI3K-AKT signalling was investigated in BCAP-deficient CD4^+^ T cell blasts. To this end, *Pik3ap1* was knocked out in primary *in vitro-*activated CD4^+^ T cells using CRISPR-Cas9-mediated gene editing via Cas9-guideRNA (gRNA) ribonucleoprotein (RNP) delivery. This approach was chosen so that, in the absence of an inducible-knockout mouse model, the role of BCAP could be interrogated in primary cells after activation and in the same physiological context in which BCAP had been identified as a p110δ interactor.

**Figure 4:**
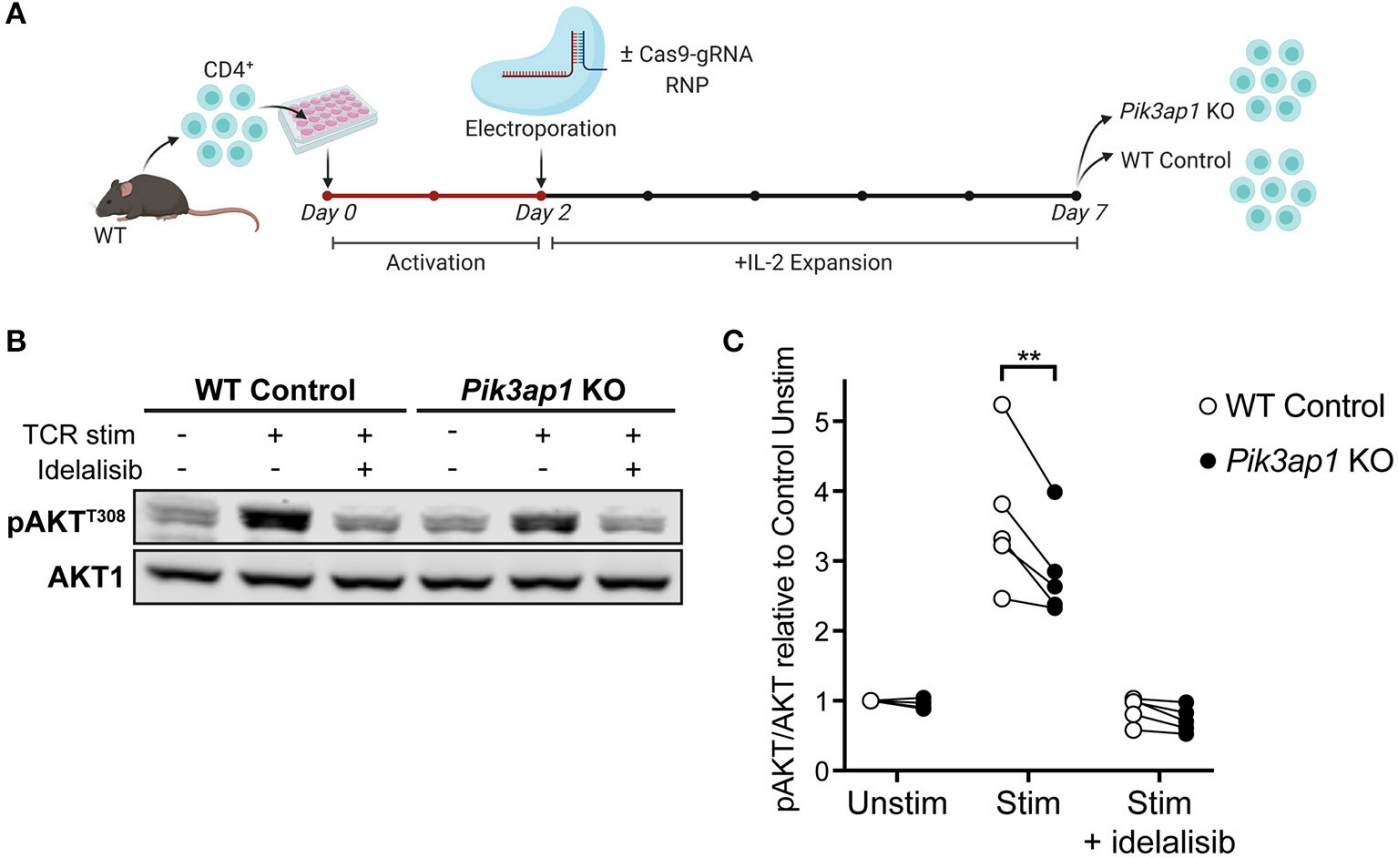
TCR-induced p110δ-AKT signalling is impaired in BCAP-deficient activated CD4^+^ T cells. **(A)** Schematic of the CRISPR-Cas9 gene editing protocol used to generate *Pik3ap1*-KO T cell blasts. Naïve CD4^+^ T cells from the lymph nodes of one WT mouse were activated *in vitro* with anti-CD3+anti-CD28 for 48 h then electroporated with (for *Pik3ap1* KO) or without (for WT Control) Cas9-gRNA RNPs targeting *Pik3ap1* exon 4. Cells were then expanded with IL-2 for 5 days. *Pik3ap1* knockout was confirmed on day 5 by sequencing and Tracking of Indels by Decomposition analysis, as described in Supplementary Figure 5. Cells were rested for 1.25 h before TCR stimulation on day 7. **(B)** Immunoblot for pAKT^T308^ in *Pik3ap1*-knockout and WT-control CD4^+^ T cell blasts following TCR stimulation for 2 min by CD3-CD4-crosslinking (+), or without stimulation (–), in the presence or absence of 200 nM idelalisib. AKT1 was detected as a loading control. Immunoblot representative of five biological repeats. **(C)** Quantification of pAKT^T308^ detected by immunoblotting in *Pik3ap1*-knockout and WT-control CD4^+^ T cell blasts, stimulated as described in **(B)**. pAKT^T308^ was normalised to the AKT1 loading control and is expressed relative to that in unstimulated WT-control cells. The lines on the spaghetti plot connect *Pik3ap1*-knockout (black circles) and WT-control (white circles) cells from the same mouse and experiment. Data are from five biological repeats. Significance was determined by a ratio paired *t*-test; **p = 0.0088.

To achieve CRISPR-Cas9-mediated gene knockout, naïve CD4^+^ cells were activated *in vitro* for 2 days before electroporation with Cas9 RNPs complexed with gRNA targeting *Pik3ap1* (Figure 4A). The cells were then expanded for 5 days in the presence of IL-2 and efficient *Pik3ap1* gene knockout (82 ± 5.6%; mean ± SEM) was confirmed by sequencing and analysis of indels (insertions and deletions) at the Cas9-target site (Supplementary Figure 5).

*Pik3ap1-*knockout cells and unedited wild-type control cells were then stimulated by CD3-CD4-crosslinking and the level of AKT phosphorylation (pAKT) was measured by immunoblotting (Figure 4B). The induction of pAKT following TCR engagement is p110δ-dependent in activated CD4^+^ cells (10, 11), such that no pAKT was induced when cells were stimulated in the presence of the p110δ-selective inhibitor, idelalisib (Figures 4B,C). Therefore, pAKT could be used as a readout of p110δ activity. Interestingly, the phosphorylation of AKT upon TCR stimulation was impaired (by 20.25 ± 4.017%; mean ± SEM) in *Pik3ap1-*knockout cells compared to wild-type control cells, indicating that BCAP positively regulates p110δ activity downstream of the TCR (Figure 4C). The ability of *Pik3ap1*-knockout cells to nevertheless induce pAKT implies that the role of BCAP is redundant, but could also reflect an incomplete loss of BCAP protein.

These results support the hypothesis that BCAP positively regulates p110δ in CD4^+^ T cell blasts following TCR engagement. Furthermore, the observations are consistent with the p110δ interactome, which revealed that multiple adaptor proteins are involved in the recruitment and activation of PI3Kδ during TCR signalling and therefore the elimination of just one of these adaptors would be expected to only partially reduce PI3Kδ signalling output. Finally, these experiments demonstrate that novel p110δ-interactors can be interrogated for their role in TCR-induced PI3K signalling in primary cells using CRISPR-Cas9 technology.

### Identification of the p110δ Interactome in Naïve CD4^+^ T Cells by AP-MS

BCAP and ICOS were identified as TCR-induced p110δ-interactors in CD4^+^ T cell blasts. However, based on the observation that BCAP is not expressed in naïve CD4^+^ T cells, and the fact that ICOS is only expressed at the plasma membrane upon CD4^+^ T cell activation (34), it is possible that p110δ employs a different set of adaptors at the TCR signalosome in naïve T cells compared to T cell blasts. Therefore, the p110δ interactome was investigated in naïve CD4^+^ T cells by AP-MS. For this, naïve CD4^+^ T cells were isolated from p110δ^AviTag^BirA^Tg^ and BirA^Tg^ mice, stimulated *in vitro* for 1 min by CD3-CD4-crosslinking, or left unstimulated, and the cell lysates were subjected to streptavidin-mediated affinity purification. The peptide samples generated from three independent experiments were then labelled with isobaric TMTs and combined for pre-fractionation followed by identification and quantification by nLC-MS/MS analysis.

**Figure 5:**
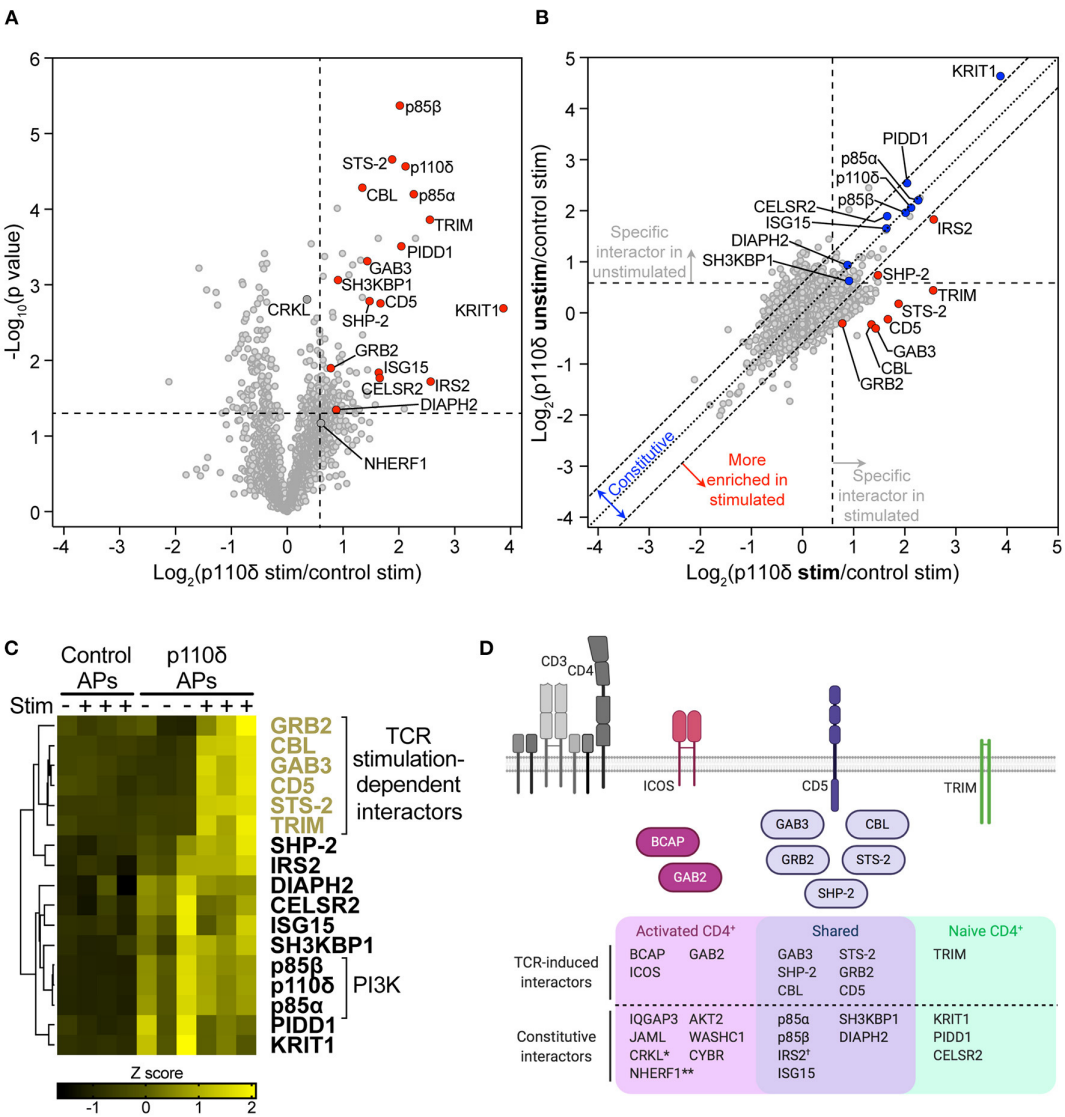
The p110δ interactome in naïve CD4^+^ T cells. **(A)** Volcano plot of proteins identified by mass spectrometry in APs from naïve CD4^+^ T cells. The plot shows the log_2_-difference in abundance of each protein in p110δ APs compared to control APs from TCR-stimulated cells from three independent repeat experiments [Log_2_(p110δ stim/control stim)] vs. the –Log_10_-*p* value, determined by a two-tailed Student's *t*-test. Cells had been stimulated for 1 min by CD3-CD4-crosslinking. The thresholds used to determine specific p110δ-interactors (upper-right quadrant) are drawn at 1.5-fold enrichment and *p* = 0.05. Proteins of interest are represented by red points. Proteins identified as specific p110δ-interactors in CD4^+^ T blasts that fall outside of the thresholds in naïve cells (CRKL, NHERF1) are filled dark grey. The full list of all proteins identified and *t*-test results can be found in Supplementary Datasheet 3. **(B)** Scatter plot comparing the abundance of identified proteins in p110δ purifications from TCR-stimulated and unstimulated naïve CD4^+^ T cells, relative to their abundance in control APs, from three independent repeat experiments. The diagonal dotted line indicates no difference between stimulated and unstimulated cells, while the diagonal dashed lines represent thresholds of 1.5-fold enrichment in either condition. The thresholds used to determine specific p110δ-interactors are drawn as vertical and horizontal dashed lines for stimulated and unstimulated cells, respectively, indicating 1.5-fold enrichment in p110δ APs compared to control APs. Specific interactors of interest are coloured blue (constitutive) and red (stimulation-induced). **(C)** Heatmap visualizing the Z score-normalised log_2_-protein abundance of specific p110δ-interactors in control and p110δ APs from unstimulated (–) and TCR-stimulated (+) naïve CD4^+^ T cells from three independent experiments. The result of hierarchical clustering of the data is represented by a dendrogram. **(D)** Diagram illustrating the proteins identified as part of the p110δ interactome in naïve CD4^+^ T cells and activated CD4^+^ T cell blasts. The Venn diagram shows p110δ-interactors common to the two cell differentiation stages (purple) and those identified in only in naïve (green) or activated (pink) CD4^+^ T cells. TCR stimulation-dependent interactors are also drawn as cartoons above the Venn diagram following the same colour scheme. NB.: *CRKL was weakly-enriched with p110δ (1.28-fold; *p* = 0.0016) in stimulated naïve T cells; **NHERF1 was inconsistently enriched with p110δ in stimulated naïve cells (1.52-fold; *p* = 0.0675); ^†^The association of IRS2 with p110δ in naïve cells was increased in TCR-stimulated compared to unstimulated cells, but was constitutive in T cell blasts.

The abundance of each identified protein was first compared between p110δ APs and control APs from TCR-stimulated p110δ^AviTag^BirA^Tg^ and BirA^Tg^ cells, respectively, in order to distinguish specific p110δ-interactors from background proteins (Figure 5A; Supplementary Datasheet 3). Proteins enriched >1.5-fold (*p* < 0.05) in p110δ purifications compared to control purifications that had not been classed as non-specific interactors in the previous APs from T cell blasts were considered to be specific p110δ-interactors in naïve CD4^+^ T cells.

As in CD4^+^ T cell blasts, p85α and p85β were identified as highly-enriched p110δ-interactors (Figure 5A). A further ten specific interactors were identified in naïve cells that had also been identified as part of the p110δ interactome in CD4^+^ T cell blasts (CBL, STS-2, GAB3, SH3KBP1, CD5, SHP-2, GRB2, IRS2, DIAPH2 and ISG15). This result indicates that p110δ forms protein complexes with a core subset of interaction partners in both naïve and previously-activated CD4^+^ T cells.

Nevertheless, ICOS, BCAP, GAB2, JAML, CYBR, IQGAP3, WASHC1 and AKT2, which associated with p110δ in blasts, were not identified in any naïve cell AP, while NHERF1 and CRKL were classed as non-specific proteins in both stimulated and unstimulated naïve cell APs (Figure 5A; Supplementary Figure 6A). This suggests that p110δ forms complexes with these proteins that may be unique to CD4^+^ T cell blasts.

Based on the fact that ICOS and BCAP are upregulated in activated T cells and BCAP is not expressed at the protein level in naïve T cells [(34); Figures 3A,B], it was possible that other differences in the p110δ-interactomes were mainly due to differential expression of the interactors. Intriguingly, analysis of publicly-available mRNA expression data from mouse CD4^+^ T cells (33) revealed that *Gab2* and *Iqgap3* are expressed at an even lower level than *Pik3ap1* in naïve CD4^+^ T cells, but are similarly upregulated in differentiated Th1, Th2 and Th17 cells (Supplementary Figures 7A,C). The gene encoding JAML (*Amica1*) is also expressed at a low level in naïve cells but is upregulated 9-fold in Th1 and Th17 cells. At the protein level, neither GAB2, IQGAP3 nor JAML were detected in the naïve CD4^+^ T cell proteome, but all three proteins are expressed upon differentiation into Th1 effector cells [Supplementary Figure 7B; (4)] Taken together, this indicates that several p110δ-binding partners are differentially expressed during T cell differentiation, which explains in part the differences in the p110δ interactomes, and also supports the theory that the regulation of p110δ recruitment downstream of the TCR differs in naïve and activated CD4^+^ T cells.

In support of this hypothesis, four proteins were identified as specific p110δ-interactors in naïve CD4^+^ T cells that had not been identified as interactors in CD4^+^ T cell blasts: the transmembrane adaptor TRIM, PIDD1, KRIT1 and the receptor CELSR2. This difference did not seem to be due to naïve cell-specific expression of PIDD1, TRIM and KRIT, as they are expressed at similar or higher levels in differentiated CD4^+^ T cells compared to naïve cells (Supplementary Figures 7A–C). However, C*elsr2* is downregulated 4-fold in Th1 and Th2 T cells compared to naïve cells (Supplementary Figure 7C), which could explain its absence from the p110δ interactome in CD4^+^ T cell blasts.

The TCR stimulation-dependency of the interactions with p110δ in naïve cells was then examined by comparing the abundance of the interactors between unstimulated and TCR-stimulated p110δ affinity purifications (Figure 5B; Supplementary Figure 6B). STS-2, GAB3, CBL, CD5, TRIM, GRB2, IRS2 and SHP-2 were more highly enriched in p110δ APs from TCR-stimulated cells than in those from unstimulated cells, indicating that their association with p110δ was induced upon TCR stimulation, as in CD4^+^ T cell blasts. These proteins did not interact specifically or significantly with p110δ in unstimulated naïve T cells (Supplementary Figure 6A) and hierarchical clustering confirmed that all but SHP-2 and IRS2 associated with p110δ in a TCR-stimulation dependent manner (Figure 5C). SHP-2 and IRS2 were associated with p110δ in unstimulated cells in some AP samples, but their interaction with p110δ was much more robust in stimulated cells (Figure 5C; Supplementary Figure 6A). The remaining interactors identified in naïve cells (SH3KBP1, DIAPH2, CELSR2, ISG15, PIDD1 and KRIT1) appeared to be constitutively associated with p110δ in both unstimulated and stimulated cells (Figures 5B,C; Supplementary Figure 6A).

Overall, comparison of the p110δ interactome in naïve and previously-activated CD4^+^ T cells strongly suggests that, although p110δ forms complexes that are common to both differentiation stages, p110δ also employs different protein adaptors in activated and naïve cells, both before and following TCR engagement (Figure 5D). Moreover, activated CD4^+^ T cells upregulate additional adaptor proteins for p110δ, namely BCAP, ICOS, GAB2, IQGAP3 and JAML.

## Discussion

In this study, we set out to uncover the proteins that link TCR engagement to PI3Kδ activation. Using transgenic mice expressing affinity-tagged p110δ in combination with quantitative proteomics, this work has elucidated the p110δ interactome in primary naïve CD4^+^ T cells and in differentiated T cell blasts. The results revealed that p110δ associates with multiple cytosolic and transmembrane adaptor proteins upon TCR engagement and is likely to form multivalent protein complexes at the TCR signalosome. Many of the identified p110δ-interactors contain YxxM motifs and are known to have the capacity to bind directly to p85 *in vitro* and in cell lines; this work now demonstrates that these interactions occur in primary T cells. In addition, this study has uncovered interactions with p110δ that were not previously known to take place in CD4^+^ T cells or to be involved in TCR-PI3K signalling, involving BCAP, GAB3, IQGAP3 and JAML. Furthermore, by comparing the p110δ interactomes in naïve and activated cells, this study has revealed that CD4^+^ T cells upregulate several p110δ adaptor proteins during differentiation. Our AP-MS approach did not enable us to identify phosphotyrosine peptides within the p110δ interactomes with high confidence. However, future phosphoproteomics experiments could be designed to identify pYxxM-containing peptides that are phosphorylated following TCR engagement in order to provide complementary insight into the regulation of PI3Kδ activity.

**BCAP** was identified as a TCR stimulation-dependent p110δ-binding partner in CD4^+^ T cell blasts through two independent MS analyses. This study also revealed that BCAP is upregulated in activated CD4^+^ T cells and is rapidly phosphorylated on tyrosine residues following TCR stimulation. Furthermore, using CRISPR-Cas9-mediated gene editing in primary activated CD4^+^ T cells, this work demonstrated that BCAP positively regulates PI3K-AKT signalling downstream of the TCR. BCAP function in previously-activated CD4^+^ T cells therefore appears analogous to its role in B lymphocytes. Upon BCR stimulation, tyrosine-phosphorylated BCAP interacts with p85 and is thought to be recruited to Igα of the BCR complex through binding to the adaptor protein NCK (29, 31). Interestingly, NCK is also able to bind to a proline-rich region in the CD3ε chain that is exposed during TCR engagement (35), so it is possible that NCK could bridge BCAP and the TCR-CD3 complex to recruit p110δ to the TCR signalosome. Previous studies have shown that BCAP-deficient DT40 B cells, which do not express the PI3K-adaptor CD19, exhibit reduced PIP_3_ production and AKT activation following BCR stimulation (29), although activation of PI3K and AKT appears to be unaffected in BCAP^−/−^ mouse splenic B cells (32). This implies that functional redundancy exists between BCAP and CD19 in B cells. Given that only a partial reduction in pAKT was observed in *Pik3ap1*-knockout activated CD4^+^ T cells, redundancy is also likely to exist between p110δ-adaptors in T cells. Indeed, the presence of multiple adaptor proteins in the p110δ interactomes following TCR stimulation supports this point.

The identification of BCAP as an adaptor and positive regulator of p110δ in previously-activated CD4^+^ T cells supports a recent study that demonstrated a role for BCAP in the clonal expansion and differentiation of effector and memory CD8^+^ T cells (36). BCAP was shown to be upregulated in *in vitro*-activated murine CD8^+^ cells and human effector/memory CD8^+^ T cells, and *Pik3ap1*^−/−^ activated CD8^+^ T cells exhibited reduced phosphorylation of AKT upon stimulation with anti-CD3. Thus, taken together with the present study, it is clear that BCAP serves as an adaptor for p110δ in previously-activated CD4^+^ and CD8^+^ T cells during TCR signalling. Furthermore, the expression of BCAP may allow for the regulation of p110δ activity by additional receptors in differentiated Th cells, given that BCAP has also been shown to be involved in the activation of the PI3K-AKT pathway downstream of the IL-1 receptor (37).

The co-stimulatory receptor **ICOS** was also identified as a TCR stimulation-dependent p110δ-interactor in CD4^+^ T cell blasts. ICOS is not expressed on resting T cells but is rapidly induced on activated T cells following TCR crosslinking (34). ICOS is known to recruit p85 from the cytoplasm to the T-cell immune synapse via a YMFM motif in its intracellular tail following either ICOS ligation or CD3-ICOS co-stimulation (38, 39). However, the p110δ interactome suggests that ICOS can recruit p85:p110δ *independently* of ICOS ligation, and solely upon TCR stimulation (CD3-CD4-crosslinking), in activated CD4^+^ T cells. This implies that ICOS is tyrosine-phosphorylated “in-trans” by the TCR signalosome. Interestingly, the related co-stimulatory receptor CD28 was not identified as a specific p110δ-interactor. The CD28 cytoplasmic tail contains a YMNM motif that when phosphorylated can directly bind to and recruit p85 in T cells (40–42). However, subsequent studies have shown that the CD28-p85 interaction is not required for PI3K activation at the immune synapse in naïve or previously-activated T cells (9, 43), and that ICOS ligation induces higher PI3K activity than CD28 ligation (38, 44, 45), which may underpin the dominant role for ICOS in PI3K signalling in T cells (39, 46).

The p110δ interactomes revealed that p110δ associates with the Gab-family member **GAB3** in both naive and previously-activated CD4^+^ T cells upon TCR engagement. However, in activated CD4^+^ T cells p110δ also interacts with **GAB2** during TCR signalling, following the upregulation of *Gab2*. GAB2 is a ubiquitously expressed and well-studied scaffolding protein, but its role in T cells has mainly been examined in cell lines. In Jurkat T cells, which are more likely to reflect the situation in T blasts rather than in naïve T cells, GAB2 is recruited to phosphorylated LAT via GRB2/GADS upon TCR engagement and is then phosphorylated by ZAP70 (47), allowing GAB2 to associate with p85 and SHP-2 (48). In contrast, GAB3 is poorly studied in T lymphocytes and thus was not previously known to be involved in TCR-PI3K signalling, even though GAB3 is most highly expressed in immune cells (49). However, it was recently suggested that GAB2 and GAB3 may perform redundant functions in immune cells, given that *Gab2/3*^−/−^ mice exhibit T cell- and macrophage-mediated colitis whereas *Gab2*^−/−^ and *Gab3*^−/−^ mice do not (49, 50). Interestingly, *in vitro-*activated *Gab2/3*^−/−^ total T cells exhibit dysregulated PI3K/AKT/mTOR signalling, with reduced pAKT^T308^ and p4EBP1 in response to IL-2 (50). This supports the conclusion that at least one of these Gab-family members regulates p110δ in activated T cells. The p110δ interactomes suggest that GAB3 is the key Gab-family protein in naïve CD4^+^ T cells. Further work in primary T lymphocytes is required to clarify the overlapping and distinct roles of GAB3 and GAB2 at different T cell differentiation stages.

In this study, p110δ was found to associate with **CD5** and **CBL** upon TCR stimulation in both naïve and previously-activated CD4^+^ T cells. The transmembrane receptor CD5 constitutively associates with the TCR and translocates to the immune synapse (51, 52) but does not contain any YxxM motifs, although *in vitro* binding assays have suggested that the p85 SH2 domains can bind to non-conventional phosphotyrosine motifs in the CD5 tail (53). In contrast, CBL is known to interact directly with p85 via a YxxM motif that is phosphorylated downstream of the TCR (54, 55), and CBL can also associate with CD5 (56–58). Furthermore, the association of CBL with CD5 is induced by CD3-CD4-crosslinking in primary mouse activated CD4^+^ T cells (57). Recent work by Blaize et al. strongly suggests that, in thymocytes, CD5 acts as a transmembrane signalling hub that is tyrosine phosphorylated upon TCR engagement and subsequently recruits a complex comprising CBL, p85, STS-2, CRKL and SH3KBP1 to the plasma membrane (58). The association of this complex with CD5 is disrupted upon loss of CBL or mutation of the predicted CBL-binding site (Y429) in the CD5 cytoplasmic tail, indicating that CBL serves as the key scaffolding protein in the assembly, likely recruiting its known binding partners, p85, STS-2, CRKL and SH3KBP1. Most interestingly, TCR-induced AKT^S473^-phosphorylation is reduced in both thymocytes and naive CD4^+^ T cells from *Cd5*^−/−^ and CD5^Y429F^ mice (58), indicating that CD5 is important for the positive regulation of PI3K signalling downstream of the TCR. Taken together, it is plausible that p110δ is recruited to CD5 at the plasma membrane via binding to phosphorylated CBL during early TCR signalling. The p110δ interactomes determined in the present study, which identified the inducible interaction of p110δ with CBL, CD5 and the purported CD5-signalling complex, support this model and imply that CD5 may serve as a key signalling hub involved in the positive regulation of PI3K in mature CD4^+^ T cells as well as in thymocytes.

It is worth noting that the detected inducible association of **STS-2** (UBASH3A) with p110δ, despite its lack of YxxM motifs, may be explained as an indirect interaction via CBL, given that STS-2 binds constitutively to CBL in CD4^+^ T cells via a direct SH3:proline-rich domain interaction (57, 59).

It was very interesting to find that p110δ associated constitutively with the multidomain adaptor proteins **CRKL** and **SH3KBP1**, as they are known to interact with p85 but are also able bind to several of the TCR-stimulation-dependent p110δ-interactors that were also identified. Specifically, while the SH3-N domain of CRKL can bind to p85 (60, 61), likely via a PxxPxK motif, the CRKL SH2 domain can interact with tyrosine-phosphorylated YxxP motifs within CBL and GAB2 (62, 63). It is therefore reasonable to hypothesise that a constitutive CRKL:p85:p110δ complex may be recruited to tyrosine-phosphorylated CBL and GAB2 following TCR stimulation, whereupon the p85 SH2 domain could interact with phosphorylated YxxM motifs within CBL (54) or GAB2 (63) to allow activation of p110δ, while CRKL simultaneously binds to CBL or GAB2.

A similar mechanism can be envisaged involving SH3KBP1, based on its constitutive interaction with p110δ in both naïve and previously-activated CD4^+^ T cells. SH3KBP1 binds via its proline-rich region and two of its three SH3 domains to p85 (64, 65), but can also interact via any of its SH3 domains with a polyproline motif present in the C-terminal tail of CBL (66). Moreover, conformational changes that occur within SH3KBP1 due to p85 binding, as well as tyrosine-phosphorylation of CBL, may subsequently allow the SH3 domains of SH3KBP1 to interact with CBL (65, 67). In T cells, the association between SH3KBP1 and CBL increases following TCR stimulation and SH3KBP1 colocalises with TCR microclusters during the first 2 min of TCR stimulation (68). It is therefore plausible that SH3KBP1:p85:p110δ complexes present in resting T cells are recruited to CBL at the TCR signalosome.

Taken together with the p110δ interactomes presented here, the evidence suggests that in resting CD4^+^ T cells p110δ may be a part of preformed complexes with CRKL and SH3KBP1 that, upon TCR stimulation, can form ternary complexes with CBL and GAB2 that are likely stabilised by their multiple inter-molecular interactions. This mechanism could facilitate the localization and activation of p110δ at the plasma membrane during early TCR signalling.

The activated CD4^+^ T cell interactome additionally revealed a constitutive association of p110δ with **IQGAP3**, the expression of which was also distinctly upregulated at the mRNA level in differentiated CD4^+^ T cell subtypes. IQGAPs are multimodular scaffolds, with three family members expressed in mammals. Little is known about IQGAP3 function, but IQGAP1, the most well-studied family member, acts as a scaffold for several signalling pathways, including components of PI3K-AKT signalling downstream of RTKs and GPCRs (69, 70). IQGAP1 scaffolds PI3K, PIPKIα, and PI(4)KIIIα together so that PI(3,4,5)P_3_ can be generated sequentially from phosphatidylinositol by each enzyme in close proximity (70). In this complex, IQGAP1 binds to p85 via its WW and IQ3 domains, which are also conserved in IQGAP3 with a high degree of homology (71). Further work is therefore warranted to investigate the IQGAP3 interaction with p110δ and the role of this protein in activated CD4^+^ T cells. IQGAP3 may enable efficient activation of the PI3K-AKT pathway in these cells by regulating the localization and proximity of PI3K-pathway components.

p110δ was also found to associate constitutively with the transmembrane glycoprotein **JAML** in previously-activated CD4^+^ T cells. JAML is proposed to function as a co-stimulatory receptor on epithelial γδ T cells by recognition of its ligand coxsackie and adenovirus receptor (CAR) on epithelial cells (72). Interestingly, resting epithelial γδ cells express very low levels of JAML at the cell surface, but upregulate its expression upon activation, and lymphoid γδ T cells require activation to express detectable levels of JAML (73). This is similar to naïve CD4^+^ αβ T cells, which upregulate *Amica1* upon differentiation into Th1 and Th17 subtypes, and may explain why JAML was detected as a p110δ-interaction partner only in activated and not naïve CD4^+^ T cells. CAR-mediated clustering of JAML on an epidermal γδ T cell line (7–17 dendritic epidermal T cells, DETCs) rapidly recruits p85 to a YMxMxPxxP motif in the JAML cytoplasmic tail (30), and ligation of JAML on these cells leads to phosphorylation of AKT (73). p85 was also found to be associated with JAML in resting cells, albeit at lower levels. It is therefore intriguing that p110δ was found to be constitutively associated with JAML in CD4^+^ T cell blasts in the present study, independently of ligand binding. It remains to be tested whether JAML ligation on previously-activated CD4^+^ αβ T cells induces PI3K-AKT signalling or potentiates TCR-induced signalling, and thus whether JAML can function as an αβ T cell co-stimulatory receptor.

The transmembrane adaptor protein **TRIM**, which associates with the TCR-CD3 complex (74, 75), was identified as a TCR stimulation-induced interactor of p110δ in only naïve CD4^+^ T cells. It has been demonstrated *in vitro* and in T-cell lines that TRIM has the ability to bind to the SH2 domains of p85 following TCR stimulation, via a YxxM motif in its cytoplasmic tail (74). TRIM appears to negatively regulate PI3K activity in order to inhibit or fine-tune TCR signalling, yet this is thought to be a redundant function shared with the transmembrane adaptors SIT and LAX, in part based on the observation that TRIM^−/−^ mice display no defects in T cell development nor function (76, 77). Nevertheless, TRIM^−/−^ CD4^+^ T cells, as well as SIT^−/−^ and LAX^−/−^ T cells, exhibit augmented TCR-induced AKT phosphorylation (76, 78, 79). The mechanism by which TRIM may negatively regulate PI3K has not been determined, but the p110δ interactomes suggest that this mechanism to restrain TCR-PI3K signalling may be predominantly at play in naïve rather than activated CD4^+^ T cells, given that TRIM was not found to be associated with p110δ in T cell blasts in this study. Consequently, it would be expected that previously-activated or effector CD4^+^ T cells could generate PIP_3_ at a higher rate than naïve CD4^+^ T cells upon TCR engagement. This theory is supported by the finding that activated CD4^+^ T cells upregulate additional, inducible adaptors for PI3K, namely BCAP, ICOS and GAB2, and therefore express a larger repertoire of proteins that could recruit and activate p110δ at the TCR signalosome, leading to greater and/or faster induction of the PI3K-AKT pathway. Indeed, it is well-known that effector T cells exhibit a lower threshold of activation than naïve T cells (80). Naïve T cells require sustained TCR-peptide-MHC engagement and TCR signalling to become activated, whereas CD4^+^ T cell blasts are activated upon engagement with as few as ten peptide-MHC complexes (81). Upregulation of additional p110δ adaptors following CD4^+^ T cell differentiation may be responsible for the lower threshold of activation exhibited by effector T cells, by reducing the number of TCRs required to be engaged by peptide-MHC to reach a threshold level of PI3K-AKT pathway activation (80).

In conclusion, this study has shed light on the proteins that connect TCR engagement to p110δ activation. The number of proteins seemingly involved in the regulation of p110δ downstream of the TCR reflect the importance of this pathway for T cell function. Nevertheless, this may pose difficulties for investigating the relative contribution of each adaptor to p110δ regulation, or even manipulating p110δ activity by targeting upstream regulators, as it is likely that redundancy exists in this system. Further work is warranted to investigate the new players in TCR and PI3K signalling that have been identified as well as the rewiring in the pathway that occurs upon T cell differentiation.

## Materials and Methods

### Mice

*Pik3cd*^Avi/Avi^ and *Rosa26*^BirA/BirA^ C57BL/6J mice were generated by T. Chessa, L.R. Stephens, and P.T. Hawkins at the Babraham Institute (26). Mice were housed under specific pathogen-free conditions within the Babraham Institute Biological Services Unit and genotyping was performed by Transnetyx (Cordova, TN). Wild-type C57BL/6J mice used for CRISPR-Cas9 experiments were purchased from The Jackson Laboratory and housed at the Cancer Research UK Cambridge Institute Biological Resources Unit. Organs were harvested when mice were aged 8–12 weeks according to Schedule 1 procedures. All animal experiments were approved by the Babraham Institute Animal Welfare and Ethical Review Body.

### CD4^+^ T Cell Activation and Expansion

Axillary, brachial, mesenteric and inguinal lymph nodes were isolated from mice and homogenised through 40 μm cell strainers with ice-cold buffer [1 × PBS (calcium and magnesium free), 0.5% BSA (A7906, Sigma), 2 mM EDTA]. Cells were washed once then resuspended at 5 × 10^6^ cells/ml in 5% FBS RPMI media [RMPI 1640 + L-glutamine (21875-034, Life Technologies), 5% heat-inactivated FBS (FBS-SA-10454/500, Labtech), 100 U/ml penicillin-streptomycin (15140-122, Life Technologies), 50 μM β-mercaptoethanol (31350-010, Life Technologies)] containing 1 μg/ml anti-CD3 (145-2C11, in-house) and incubated in 6-well plates for 48 h at 37°C and 5% CO_2_. Cells were then pooled, washed and resuspended at 0.5 × 10^6^ cells/ml in 5% FBS RPMI media containing 20 ng/ml recombinant human IL-2 (200-02, PeproTech). Cells were incubated for 5 days at 37°C and 5% CO_2_ with supplementation of IL-2 and media every 2 days to maintain a cell concentration of 1 × 10^6^ cells/ml. To purify CD4^+^ T cell blasts, cells were washed in 5% FBS RPMI then resuspended in 1 ml of FITC-conjugated antibody cocktail (anti-I-A^b^ MHC class II (553551, BD Biosciences), anti-CD19 (115506, BioLegend), anti-CD49b (108906, BioLegend), and anti-CD8a (100706, BioLegend); all at 1:500 dilution in PBS/BSA/EDTA buffer) per 10^8^ cells and incubated on ice for 30 min in the dark. CD4^+^ T blasts were then isolated by negative selection using anti-FITC microbeads (130-048-701, Miltenyi Biotec) and LS magnetic columns (Miltenyi Biotec) according to the manufacturer's protocol.

### Naïve CD4^+^ T Cell Isolation

Axillary, brachial, mesenteric, and inguinal lymph nodes were isolated from mice and homogenised through 40 μm cell strainers with ice-cold buffer [1 × PBS (calcium and magnesium free), 0.5% BSA (A7906, Sigma), 2 mM EDTA]. Cells were washed once then resuspended in 1 ml of FITC-conjugated antibody cocktail per 10^8^ cells [anti-I-A^b^ MHC class II (553551, BD Biosciences), anti-CD19 (115506, BioLegend), anti-CD49b (108906, BioLegend), anti-CD8a (100706, BioLegend), anti-CD69 (104506, Biolegend), anti-CD25 (102006, BioLegend), anti-B220 (103206, BioLegend), and anti-CD11b (101205, BioLegend); all at 1:500 dilution in PBS/BSA/EDTA buffer] and incubated on ice for 30 min in the dark. Naïve CD4^+^ T cells were then isolated by negative selection using anti-FITC microbeads (130-048-701, Miltenyi Biotec) and LS magnetic columns (Miltenyi Biotec) according to the manufacturer's protocol.

### TCR Stimulation

Before TCR stimulation, purified CD4^+^ T cells (naïve or blasts) were resuspended at 4 × 10^6^ cells/ml in 0.5% FBS RPMI media and rested for 75 min at 37°C. For experiments involving pharmacological inhibition of p110δ, the media was supplemented with 200 nM DMSO (D8418, Sigma) or 200 nM idelalisib (AstraZeneca, in-house). Cells were collected by centrifugation then incubated at 2 × 10^7^ cells/ml in 0.5% FBS RPMI containing 4 μg/ml anti-CD3 (100314, BioLegend) and 4 μg/ml anti-CD4 (100520, BioLegend), with or without 200 nm DMSO/idelalisib, for 30 min on ice. Cells were washed by centrifugation and resuspended in ice-cold 0.5% FBS RPMI (±DMSO/idelalisib) at 2 × 10^7^ cells/ml. Cells were incubated at 37°C for 2 min before addition of 13 μg/ml goat anti-Armenian Hamster IgG (127-005-099, Jackson ImmunoResearch) and further incubation at 37°C for the times indicated in accompanying figure legends. Cells were immediately centrifuged at 800 × g for 2 min at 4°C and then resuspended in ice-cold lysis buffer (1% IGEPAL CA-630, 50 mM HEPES, 150 mM NaCl, 1 mM EDTA, 1 mM EGTA, 25 mM NaF, 10 mM Iodoacetamide, 2.5 mM sodium pyrophosphate, 5 mM β-glycerophosphate, 5 mM sodium orthovanadate, and 5 mM Proteoloc Protease Inhibitor Cocktail (Expedeon Ltd); 100 μl per 1 × 10^7^ cells) and incubated on ice for 10 min. For affinity purification experiments, lysates were cleared by ultracentrifugation at 96,416 × g for 5 min at 4°C. For IP or immunoblotting-only experiments, lysates were cleared by centrifugation at 20,000 × g for 10 min at 4°C.

### Naïve B Cell Isolation and BCR Stimulation

Mouse spleens were homogenised through 40 μm cell strainers and red blood cells were lysed by incubation with Red Blood Cell Lysing Buffer (R7757, Sigma-Aldrich) for 5 min at room temperature. Naïve B cells were isolated by negative selection using a mouse B Cell Isolation Kit (130-090-862, Miltenyi Biotec) and LS magnetic columns (Miltenyi Biotec) according to the manufacturer's protocol.

Before BCR stimulation, purified naïve B cells were resuspended at 4 × 10^6^ cells/ml in 0.5% FBS RPMI media and rested for 75 min at 37°C. Cells were collected by centrifugation then incubated at 2 × 10^7^ cells/ml in 0.5% FBS RPMI containing 50 μg/mL goat anti-mouse IgM [F(ab′)_2_ fragment; 115-036-075, Jackson ImmunoResearch] for 30 min on ice. B cells were then stimulated by incubation at 37°C for 2.5 min. Cells were immediately centrifuged at 800 × g for 2 min at 4°C and then resuspended in ice-cold lysis buffer (100 μl per 1 × 10^7^ cells), incubated on ice for 10 min and cleared by centrifugation at 20,000 × g for 10 min at 4°C.

### Affinity Purification

Fresh, cleared lysates from 1 × 10^8^ T cell blasts or 0.19 × 10^8^ naïve T cells were immediately incubated with 60 or 19 μl, respectively, of washed streptavidin-conjugated Dynabeads® (M-280 Streptavidin, 11205D, Invitrogen) for 30 min on a rotating wheel at 4°C. Beads were separated using a magnetic tube rack and “post” lysates were retrieved. Beads were washed three times with 1 ml ice-cold lysis buffer then resuspended in 1 × NuPAGE LDS Sample Buffer (NP0007, Invitrogen) and boiled at 95°C for 5 min. Beads were allowed to return to room temperature then separated on the magnetic rack and eluates were retrieved to low-protein binding microcentrifuge tubes and snap frozen in liquid nitrogen.

### Immunoblotting

Cell lysate samples were denatured and reduced in 1 × NuPAGE LDS Sample Buffer (NP0007, Invitrogen) with 20 mM DTT at 70°C for 10 min. Lysate, AP and IP samples were subjected to SDS-PAGE on NuPAGE 4–12% Bis-Tris Gels (Invitrogen) and proteins transferred onto PVDF membranes using the XCell SureLock Mini-Cell Electrophoresis System and XCell II Blot Module (Invitrogen) according to the manufacturer's instructions. Membranes were blocked in 5% BSA (w/v) TBS-Tween and incubated with the following primary antibodies overnight at 4°C: anti-p110δ (sc-7176, Santa Cruz), anti-pan-p85 (06-195, Millipore), anti-phosphotyrosine (05-321, Millipore), anti-BCAP (generous gift from T. Kurosaki), anti-pAKT T308 (4056, Cell Signaling Technology), anti-AKT1 (2967, Cell Signaling Technology); all in 5% BSA TBST. Washed membranes were incubated with the following secondary antibodies for 1 h at room temperature: goat anti-rabbit IRDye® 680RD (926-68071, LI-COR) or goat anti-mouse IRDye® 800CW (926-68070, LI-COR); in 5% BSA-TBST. For detection of pAKT and AKT1, membranes were incubated with both primary or secondary antibodies simultaneously. Membranes were imaged using an Odyssey® CLx Imaging System (LI-COR) and images were quantified using Image Studio Lite Version 5.2 (LI-COR). Statistical analyses as detailed in figure legends were performed in GraphPad Prism (GraphPad Software, Inc.).

### Peptide Sample Preparation for MS Analysis

Denatured affinity purification samples were subjected to SDS-PAGE on Bolt 10% Bis-Tris Plus Gels (Invitrogen) until samples fully entered the gel, or, for the targeted gel slice analysis, samples were separated fully for 1 h on a 4–12% Bis-Tris Gel (Invitrogen). Proteins were visualised with Imperial Protein Stain (ThermoFisher Scientific) and excised, or just the region encompassing 110–120 kDa was excised using the 100 kDa marker and p110δ (119.7 kDa) protein band as reference.

Coomassie dye was removed from 1 × 1 mm gel pieces by sequential washes in 12.5 mM ammonium bicarbonate (AMBI) and 50% acetonitrile (ACN) with 20 min sonication. Samples were dehydrated with 100% ACN for 10 min with sonication then rehydrated and reduced with 10 mM DTT in 25 mM AMBI for 60 min at 50°C. Samples were alkylated with 50 mM iodoacetamide in 25 mM AMBI for 60 min at room temperature with shaking in the dark. Reduced and alkylated samples were washed with 25 mM AMBI followed by dehydration with 100% ACN and 10 min sonication, repeated twice, and drying in a vacuum centrifuge. Gel pieces were rehydrated for 20 min at room temperature with trypsin solution (10 ng/μl trypsin in 50 mM triethylammonium bicarbonate with 0.1% octylglucoside) then digested overnight at 30°C in a total volume of 50 μl.

For quantitative MS analyses, samples were subjected to in-gel tandem mass tag (TMT) labelling. Each 0.2 mg aliquot of TMT10plex Isobaric Label Reagent (ThermoFisher Scientific) was resuspended in 20 μl 100% ACN and added to individual digested samples, followed by incubation for 1 h at room temperature with sonication and vortexing every 15 min. Labelling was quenched with 0.3% hydroxylamine for 30 min at room temperature with sonication. TMT-labelled peptides were extracted from gel pieces with 100% ACN in a series of 3 washes with 25 μl 100% ACN and 10 min sonication per wash. Final extracts from all 10 samples were combined in a 1:1 ratio and dried in a vacuum centrifuge. The dried sample was resuspended in 16 μl 0.2% NH_3_ for high-pH reversed-phase fractionation on the UltiMate 3000 system (ThermoFisher Scientific). An in-house made microcolumn [0.53 × 200 mm packed with ReproSil-Pur C18-AQ resin, 3 μm particle size (Dr. Maisch, Germany)] was used for peptide separation at 20 μl/min following a linear gradient from 0 to 40% ACN over 30 min. Eluted peptides were manually collected into 1-min fractions and stored at −80°C until nLC-MS/MS analysis.

### nLC-MS/MS Analysis

TMT-labelled peptide-enriched fractions were analysed by nano-liquid chromatography-tandem mass spectrometry (nLC-MS/MS) using an EASY-nLC System (ThermoFisher Scientific) interfaced via a nano-electrospray ion source onto a Q Exactive Plus Hybrid Quadrupole-Orbitrap Mass Spectrometer (ThermoFisher Scientific). Peptide separation was performed using an in-house made pre-column (100 μm ID fritted fused silica packed with POROS 20 R2 Reversed-Phase Resin) and analytical column [75 μm ID × 15 cm fused silica capillary with integrated emitter (New Objective) packed with ReproSil-Pur C18-AQ resin (2.1 μm; Dr. Maisch, Germany)]. Half of each fraction was used. Samples were loaded onto the pre-column in solvent A (0.1% formic acid) at a flow rate corresponding to 60 bar. Peptides were then separated over the analytical column at a flow rate of 300 nl/min following a linear gradient from 5 to 35% solvent B (100% ACN) over 60–90 min. Eluted peptides were ionised by applying a 1.6–1.7 kV voltage and introduced to the mass spectrometer as gas-phase ions. MS1 scans were only triggered when a threshold of 3e6 ions or 50 ms was reached and were acquired in the Orbitrap mass analyser with a mass resolution of 70,000 and a scan range of m/z 350–1,800. The 12 most intense ions from each MS1 spectrum were isolated in the quadrupole mass analyser using a 1.2 m/z window and fragmented using higher-energy collisional dissociation with normalised collision energy of 30 and 35. MS2 scans of fragment ions were only triggered when a threshold of 5e4 ions or 500 ms was reached and were acquired in the Orbitrap mass analyser with a mass resolution of 35,000 and mass range starting at 100 m/z. Fragmented ions were excluded from repeated analysis for 30 s. MS and MS2 spectra were recorded in Xcalibur 3.0 (ThermoFisher Scientific).

Label-free peptide samples from the targeted gel slice experiment were analysed using the same equipment and slightly different parameters as detailed in Supplementary Methods.

### Protein Identification and Quantification

Acquired MS data were processed in Proteome Discoverer 2.1 (ThermoFisher Scientific). Spectra from all fractions were combined and searched against the mouse UniProt reference proteome (UP000000589, 52,026 entries) and Global Proteome Machine database of common contaminants (57 entries) using Mascot search engine. Trypsin was set as the specific protease with a maximum of two missed cleavage sites allowed. Peptide mass tolerance was set to 10 ppm and fragment mass tolerance was set to 20 mmu. Carbamidomethylation of cysteine and TMT modification of the peptide N-terminus and lysine side chains were set as fixed modifications. Oxidation of methionine was set as a variable modification. Only high confidence peptides were used, with a 0.01 target peptide FDR, and a minimum of 1 unique peptide was required for successful protein identification. For TMT-labelled datasets, reporter ion intensities were extracted from MS2 spectra that exceeded an average reporter signal-to-noise threshold of 10 with a co-isolation interference lower than 50%. Reporter ion intensities were normalised to the total peptide amount per channel within Proteome Discoverer 2.1. For the label-free gel-slice dataset, precursor ions area detector node was used for label-free protein quantitation within Proteome Discoverer.

The mass spectrometry proteomics data have been deposited to the ProteomeXchange Consortium (http://proteomecentral.proteomexchange.org) via the PRIDE partner repository (82) with the dataset identifier PXD022607.

### Interactome Analysis

Quantitative MS data was analysed in Perseus Version 1.6.7.0 (83, 84). Abundance values for high-confidence proteins, excluding contaminants, that were identified and quantified in all ten affinity purification samples were first log_2_-transformed in Perseus. For the T blast interactome, data from only nine affinity purifications could be analysed as one sample (p110δ AP, unstimulated, repeat 1) was labelled with a faulty TMT label. To distinguish specific interactors from non-specific background, two-tailed Student's *t*-tests were performed within Perseus to compare the mean abundance of each protein in TCR-stimulated control purifications (*n* = 3) with its mean abundance TCR-stimulated p110δ purifications (*n* = 3). Generated *p*-values were also corrected for multiple hypothesis testing using the permutation-based false discovery rate (FDR) method in Perseus, with a threshold of 0.21 (naïve dataset) or 0.31 (blast dataset), to generate q values (adjusted *p*-values) (84). Full *t*-test results and *q* values can be found in Supplementary Datasheets 1, 3. Specific interactors were classed as proteins with a ≥1.5-mean-fold-enrichment in p110δ purifications compared to control purifications and a *p*-value ≤0.05. Moderately-conservative thresholds were chosen to enable consideration of a higher number of potential true interactors, with the accompanying risk of potential false positives mitigated during analysis by considering the biological relevance of proteins (85). When determining proteins of interest among the specific interactors, proteins with irrelevant subcellular localization (Nuclear, Mitochondrial, ER, Golgi, Centrosomal) or function (Metabolic Process, HSP90 Co-chaperone, Unknown function) with respect to TCR-PI3K signalling [according to UniProt entries, Gene Ontology (GO) annotations and published literature] were not considered as proteins of interest. In addition, proteins in the naïve T cell dataset that passed the enrichment ≥1.5-fold and *p*-value ≤0.05 thresholds but were classified as non-specific in the T blast dataset were excluded to reduce the risk of false positives, at the cost of potentially missing true interactors that were specific to naïve cells. Reassuringly, 79.5% of these non-specific exclusions were enriched <2-fold in naïve cell p110δ APs compared to control APs. Full lists of specific interactors, detailing proteins of interest and irrelevant exclusions, can be found in Supplementary Datasheets 1, 3.

Log_2_-fold change and –log_10_-*p* values were exported from Perseus to GraphPad Prism to generate volcano and scatter plots. For heatmap visualization of protein abundance, log_2_-transformed abundance values for proteins of interest in all 9 or 10 affinity purification samples were first normalised in Perseus using Z-score transformation. Unsupervised hierarchical clustering was then performed on rows using Euclidean distance and average linkage. Z-score-transformed values and dendrograms were exported to GraphPad Prism to generate heatmaps.

### Immunoprecipitation

Fresh, cleared lysates from 1 × 10^7^ cells were incubated with 2 μl rabbit polyclonal anti-mouse-BCAP (a generous gift from T. Kurosaki) or 10 μl normal rabbit IgG (2729, Cell Signaling Technology) for 45 min at 4°C, followed by addition of 50 μl of 50% protein-A-Sepharose® bead slurry (P3391, Sigma) and further incubation for 30 min at 4°C. Beads were washed three times with lysis buffer at 4°C then resuspended in 25 μl 2 × NuPage LDS sample buffer (Invitrogen) with 20 mM DTT and proteins were eluted from the beads by incubation at 95°C for 5 min.

### CRISPR-Cas9 Editing of Primary Activated T Cells

Axillary, brachial, mesenteric, and inguinal lymph nodes from individual wild-type mice were homogenised through 40 μm cell strainers and CD4^+^ T cells were purified from single-cell suspensions by negative selection using a CD4^+^ T Cell Isolation Kit (130-104-454, Miltenyi Biotec) and LS magnetic columns (Miltenyi Biotec) according to the manufacturer's protocol. Purified CD4^+^ cells were activated for 48 h at 1 × 10^6^ cells/ml in 5% FBS RPMI media in 24-well plates coated with 1 μg/ml anti-CD3 (100314, BioLegend) and 2 μg/ml anti-CD28 (102112, BioLegend). Following activation, 1 × 10^6^ cells were washed in 1 × PBS and resuspended in 5.5 μl of Neon Buffer R (Invitrogen). Alt-R® CRISPR-Cas9 crRNA targeting exon 4 of *Pik3ap1* (5'-CAGTGTGCCTTCCACCCGGA-3') and Alt-R® CRISPR-Cas9 tracrRNA [both purchased from Integrated DNA Technologies (IDT) and resuspended in Nuclease-Free IDTE Buffer] were mixed in equimolar amounts, annealed at 95°C for 5 min, then cooled to 20°C to generate 50 μM duplex gRNA. RNP complexes were formed by incubating 10 μg TrueCut™ Cas9 Protein v2 (Invitrogen) with 4.9 μl gRNA in a total volume of 9.5 μl Buffer R for 15 min at room temperature. 1 × 10^6^ cells in Buffer R were mixed with RNP complexes along with 0.144 μM carrier ssDNA and then electroporated in 10 μl Neon tips (Invitrogen) using the Neon Transfection System (programme #24; voltage: 1,600 V, width: 10 ms, pulses: 3; Invitrogen). For unedited wild-type controls, cells were electroporated with corresponding volumes of Buffer R and IDTE Buffer. Duplicate electroporations were combined in 1 mL of 5% FBS RPMI media containing 20 ng/ml recombinant human IL-2 (PeproTech) and cultured in 24-well plates for 5 days at 37°C and 5% CO_2_, with supplementation of IL-2 and media every 24 h for the first 3 days to maintain a cell concentration of ~1 × 10^6^ cells/ml. Genomic DNA was extracted from cell aliquots collected at 72 h post-electroporation and a ~1,000 bp region spanning the *Pik3ap1* Cas9 cleavage site was PCR amplified, purified and Sanger sequenced. To estimate insertion-deletion (indel) frequencies, Sanger sequencing chromatograms of PCR amplicons were analysed using TIDE version 1.1.2 according to Brinkman et al. (86) and Brinkman and van Steensel (87) (https://tide.nki.nl). Amplicons generated from CRISPR-Cas9-edited cells were compared to amplicons from unedited wild-type cells.

## Data Availability Statement

The datasets presented in this study can be found in online repositories. The names of the repository/repositories and accession number(s) can be found in the article/Supplementary Material.

## Ethics Statement

The animal study was reviewed and approved by The Babraham Institute Animal Welfare and Ethical Review Body.

## Author Contributions

DHL designed and conducted the experiments, analysed the data and wrote the manuscript. KW and DO performed the nLC-MS/MS including database searches. TC, PTH, and LRS generated the AviTag mice and provided technical advice. DHL, STB, KH, and KO designed the study and interpreted the results. KO conceived the study and revised the manuscript. All authors contributed to the article and approved the submitted version.

## Conflict of Interest

STB and KH were employed by the company AstraZeneca. The remaining authors declare that the research was conducted in the absence of any commercial or financial relationships that could be construed as a potential conflict of interest.
