# Supplementary material for: PI3Kδ Forms Distinct Multiprotein Complexes at the TCR Signalosome in Naïve and Differentiated CD4+ T Cells
Source: Front Immunol. 2021 Mar 8;12:631271. doi: 10.3389/fimmu.2021.631271 (PMC7982423; doi:10.3389/fimmu.2021.631271)
Supplement: Supplementary file 1 [file Table_1.DOCX]

**Supplementary Table 1.** Direct interaction of identified p110δ-interactors with p85 or other identified p110δ-interactors. References provide evidence of direct, binary interaction (unless specified) and the domains/motifs required, but are not exhaustive.

| **Protein Name** | **UniProt Accession** | **Direct binding to p85** | **Binding to other p110δ interactors** |
| --- | --- | --- | --- |
| **ICOS** | Q9WVS0 | Harada *et al*, 2003 (GST-ICOS + Jurkat lysates; Y181F)  Nurieva *et al*, 2007 (*in vitro* GST PDs)  Gigoux *et al*, 2009 (co-IP CD4+ T blasts)  YxxM required | n/a |
| **GAB3** | Q8BSM5 | Direct binding to p85 is not yet confirmed:  Wolf *et al*, 2002; Jia *et al*, 2017 – co-IP only.  NB. Three YxxM motifs are conserved from GAB2 (see GAB2). | **GRB2** – (GST pull downs, SH3:PRD, Wolf *et al*, 2002)  **SHP-2** – (co-IP only, Wolf *et al*, 2002, but two known SHP-2 binding motifs (YLXL/YVXV) are conserved in GAB2 and GAB3)  NB. GAB3 lacks CRKL-binding site found in GAB2. |
| **BCAP** | Q9EQ32 | Okada *et al*, 2000 - YxxM required | n/a |
| **GRB2** | Q60631 | Wang *et al*, 1995; Weinger *et al*, 2008  Direct, SH3:PR | **CBL** – Meisner *et al*, 1995 (GRB2 SH3; constitutive)  **SH3KBP1** – Borinstein *et al*, 2000 (NB. rat SETA)  **GAB2, GAB3**: Lock *et al*, 2000; Wolf *et al*, 2002  **IRS2** – see IRS2 |
| **STS-2** | Q3V3E1 | n/a | **CBL**- Feshchenko *et al*, 2004 |
| **CBL** | P22682 | Hunter *et al*, 1999  Gelkop *et al*, 2001  Thien *et al*, 2010  Requires YxxM (Y737 in mouse) | **STS2** – Feshchenko *et al*, 2004  **CD5** – Blaize *et al*, 2020 (implied by Y429 mut)  **CRKL** – Reedquist *et al*, 1996 (co-IP; CRK-SH2 tested)  **SH3KBP1** – Kowanetz *et al*, 2003  **GRB2** – Meisner *et al*, 1995 (GRB2 SH3; constit) |
| **SHP-2** | P35235 | n/a | **GAB2** – Gu *et al*, 1998; Crouin 2001  **GAB3** – co-IP, Wolf *et al*, 2002. See GAB3.  **IRS2** – see IRS2 |
| **GAB2**  **(p97)** | Q9Z1S8 | Gu *et al*, 2000 (co-IP requires YxxMs);  Crouin *et al,* 2001 (Y2H) | **GRB2** – Lock *et al*, 2000  **SHP-2** – Gu *et al*, 1998; Crouin *et al*, 2001  **CRKL** – Crouin *et al*, 2001 (Y2H with CRKL SH2) |
| **CD5** | P13379 | n/a | **CBL** – Blaize *et al*, 2020 (implied by Y429 mut) |
| **JAML** | Q80UL9 | Verdino *et al*, 2010 (implied by Y314 mutation and co-IP) | n/a |
| **CYBR** | Q91VY6 | n/a | n/a |
| **IQGAP3** | F8VQ29 | n/a | n/a |
| **IRS2** | P81122 | Sun *et al*, 1997 (p85-nSH2 pull down)  6 of the 9 YxxM motifs in IRS-2 are conserved in IRS-1 (White, 1997), including 4 sites (Y538, Y649, Y671, Y758) that in IRS-1 can interact with p85-SH2s (Yonezawa *et al*, 1992; Sun *et al*, 1993). | **GRB2** – Argetsinger *et al*, 1996 (co-IP); Sun *et al*, 1997 (GRB2-SH2 pull down); Bisson *et al*, 2001 (AP-MS).  **SHP-2** – IRS-2 phospho-peptides can bind to SHP-2 (pull down-MS, Hanke & Mann, 2009); however, the SHP-2-nSH2 domain appears to bind only to IRS-1 (co-IP, Sun *et al*, 1997). SHP-2 binding sites (YIDL/YIAI, YASI) are conserved between IRS-1 and IRS-2. |
| **ISG15** | Q64339 | n/a | n/a |
| **NHERF1** | P70441 | n/a | n/a |
| **SH3KBP1** | Q8R550 | Gout *et al*, 2000; Borthwick *et al,* 2004 | **CBL** – Kowanetz *et al*, 2003  **GRB2** – Borinstein *et al*, 2000 (NB. rat SETA) |
| **WASHC1** | Q8VDD8 | n/a | n/a |
| **CRKL** | P47941 | Sattler *et al*, 1997; Gesbert *et al*, 1998 (via CRKL N-SH3) | **CBL** – Reedquist *et al*, 1996 (co-IP; CRK-SH2 tested)  **GAB2** – Crouin *et al*, 2001 (Y2H with CRKL SH2) |
| **AKT2** | Q60823 | n/a | n/a |
| **DIAPH2** | O70566 | n/a | n/a |
| **TRIM** | Q3UU67 | Bruyns *et al*, 1998. Requires YxxM. | n/a |
| **CELSR2** | Q9R0M0 | n/a | n/a |
| **PIDD1** | Q9ERV7 | n/a | n/a |
| **KRIT** | Q6S5J6 | n/a | n/a |

**Supplementary Table 2.** Identified p110δ-interactors containing at least one YxxM motif within their amino acid sequence.

| **Protein Name** | **UniProt Accession** | **Location of YxxM motifs (murine sequence)** |
| --- | --- | --- |
| **ICOS** | Q9WVS0 | **181 - 184:** YmfM |
| **GAB3** | Q8BSM5 | **395 - 398:** YvpM  **416 - 419:** YipM  **515 - 518:** YiqM |
| **BCAP** | Q9EQ32 | **264 - 267:** YtdM  **420 - 423:** YesM  **445 - 448:** YesM  **460 - 463:** YveM |
| **GRB2** | Q60631 | **52 - 55:** YieM |
| **CBL** | P22682 | **369 - 372:** YceM  **737 - 740:** YeaM |
| **SHP-2** | P35235 | **380 - 383:** YgvM |
| **GAB2**  **P97** | Q9Z1S8 | **441 - 444:** YvpM  **465 - 468:** YipM  **573 - 576:** YvpM |
| **JAML** | Q80UL9 | **355 - 358:** YmtM |
| **IQGAP3** | F8VQ29 | **1269 - 1272:** YsdM |
| **IRS2** | P81122 | **536 - 539:** YgyM  **538 - 541:** YmsM  **594 - 597:** YtlM  **649 - 652:** YmpM  **671 - 674:** YmpM  **734 - 737:** YmrM  **758 - 761:** YlnM  **814 - 817:** YvlM  **1061 - 1064:** YteM |
| **AKT2** | Q60823 | **177 - 180:** YyaM  **341 - 344:** YemM |
| **TRIM** | Q3UU67 | **80 - 83**: YeqM |
| **CELSR2** | Q9R0M0 | **436 - 439**: YsiM |
