# Supplementary material for: PI3Kδ Forms Distinct Multiprotein Complexes at the TCR Signalosome in Naïve and Differentiated CD4+ T Cells
Source: Front Immunol. 2021 Mar 8;12:631271. doi: 10.3389/fimmu.2021.631271 (PMC7982423; doi:10.3389/fimmu.2021.631271)
Supplement: Supplementary file 5 [file Image_1.PDF]

Supplementary Materials

This file contains Supplementary Figures 1 to 7 and Supplementary Materials & Methods.

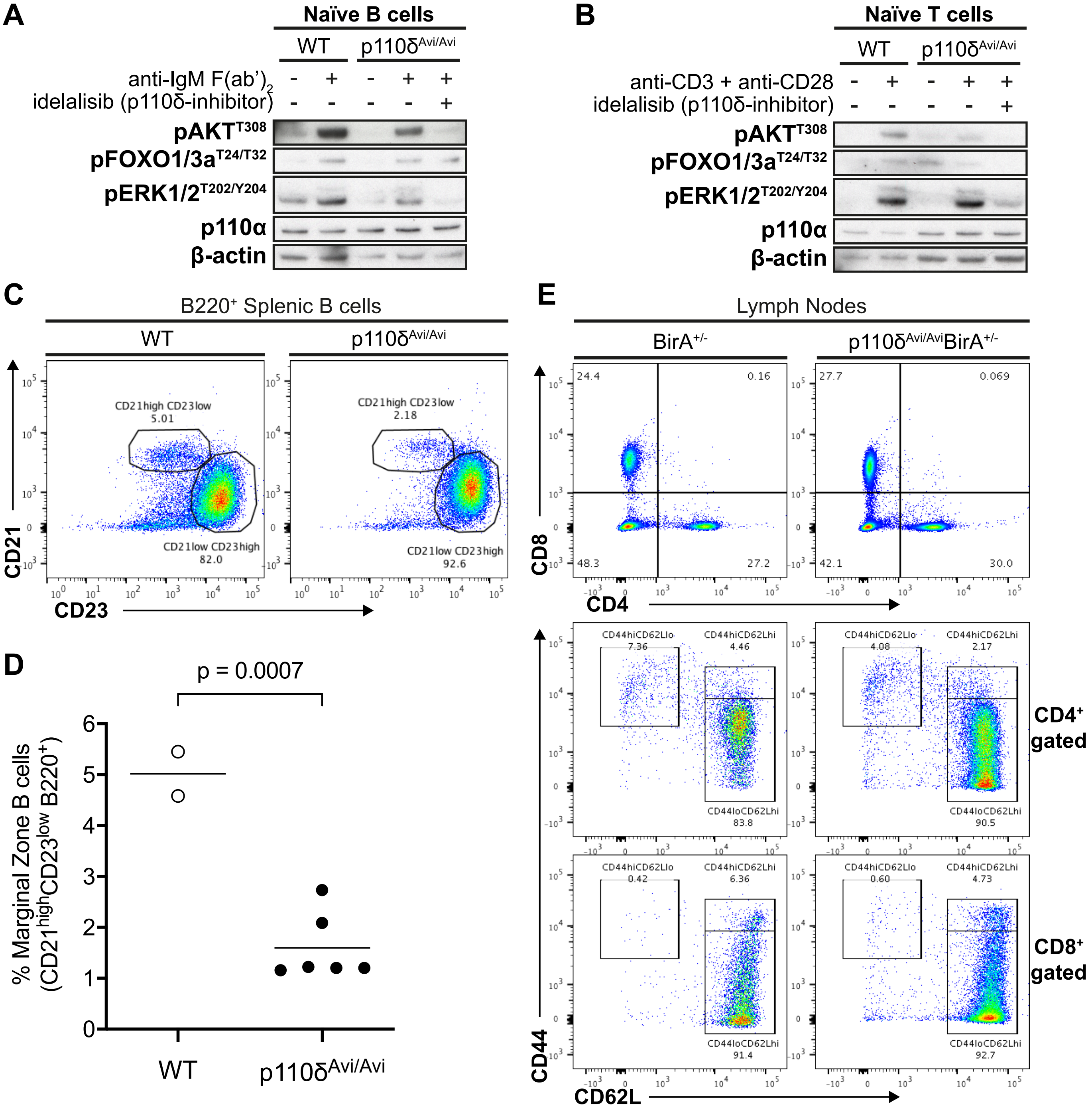

Supplementary Figure 1. AviTagged-p110δ is kinase-active in primary lymphocytes.

(A) Immunoblot of signalling events downstream of p110δ in naïve B cell lysates from wild-type or *Pik3cd*$^{Avi/Avi}$ (p110δ$^{Avi/Avi}$) mice following BCR stimulation. Naïve splenic B cells were stimulated with anti-mouse IgM F(ab')$_2$ for 3 minutes, in the presence or absence of the p110δ-selective inhibitor idelalisib at 200 nM, or left unstimulated (-). Immunoblot from one experiment respresentative of three independent experiments.

(B) Immunoblot of signalling events downstream of p110δ in naïve T cell lysates from wild-type or p110δ$^{Avi/Avi}$ mice following TCR stimulation. Naïve total T cells from lymph nodes were stimulated by anti-CD3 plus anti-CD28-crosslinking for 3 minutes, in the presence or absence of the p110δ inhibitor idelalisib at 200 nM, or left unstimulated (-). Immunoblot from one experiment respresentative of three independent experiments. **(A,B)** Immunoblots were probed with anti-phosphorylated-AKT (pAKT^{T308}), anti-phosphorylated-FOXO1/3a (pFOXO1/3a$^{T24/T32}$), anti-phosphorylated-ERK1/2 (pERK1/2$^{T202/Y204}$), anti-p110α, and anti-β-actin as loading control. BCR- and TCR-induced pAKT, pFOXO1/3a and pERK1/2 was inhibited in the presence of idelalisib in p110δ$^{Avi/Avi}$ cells, indicating that AviTagged-p110δ is kinase-active, albeit its activity may be partially impaired, as evidenced by the reduced level of pAKT induced in p110δ$^{Avi/Avi}$ cells compared to WT cells.

(C) Flow cytometric analysis of B lymphocytes (B220$^+$) from the spleens of representative wild-type (WT) and p110δ$^{Avi/Avi}$ mice. The percentages of Marginal Zone B cells (CD21^{high}CD23^{low}) and Follicular B cells (CD21^{low}CD23^{high}) are indicated next to their respective gate. Data are from one female mouse aged 9 weeks of each genotype, representative of two (WT) or six (p110δ$^{Avi/Avi}$) mice. Marginal Zone B cell development is p110δ-dependent, and the Marginal Zone B cell population is near absent in mice expressing kinase-inactive p110δ^{D910A} (Clayton *et al*, 2002; Okkenhaug *et al*, 2002).

(D) Scatter plot showing the percentage of Marginal Zone B cells (CD21^{high}CD23^{low} B220$^+$) in WT (n = 2) and p110δ$^{Avi/Avi}$ (n = 6) female mice aged 9 weeks. The horizontal bars indicate the mean. Each point represents one mouse. p values were determined by an unpaired t test. The reduced percentage of Marginal Zone B cells in the spleens of p110δ$^{Avi/Avi}$ mice compared to wild-type mice suggests that AviTagged-p110δ kinase activity may be impaired, but p110δ is still functional.

(E) Flow cytometric analysis showing normal CD4$^+$ and CD8$^+$ T cell ratios in the lymph nodes of representative p110δ$^{Avi/Avi}$BirA$^{+/-}$ and BirA$^{+/-}$ (control) mice, as well as the proportions of naïve (CD44^{lo}CD62L^{hi}), central memory (CD44^{hi}CD62L^{hi}) and effector/effector memory (CD44^{hi}CD62L^{lo}) cells within these CD4$^+$ and CD8$^+$ populations. Data are representative of two mice from each genotype. Reduced CD44 expression in p110δ$^{Avi/Avi}$BirA$^{+/-}$ naïve T cells suggests that p110δ kinase activity is slightly reduced, given that CD44 expression is reduced to a greater extent in T cells from mice that express kinase-inactive p110δ^{D910A} (Okkenhaug *et al*, 2002).

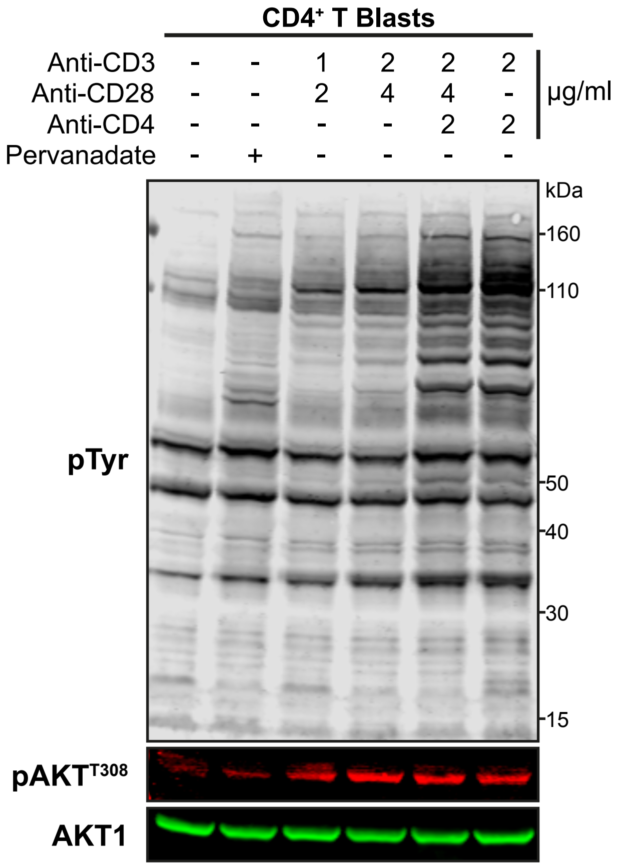

Supplementary Figure 2. Greatest cellular protein tyrosine phosphorylation in CD4$^+$ T cell blasts is achieved by TCR stimulation via anti-CD3 plus anti-CD4 crosslinking.

Immunoblot of whole cell lysates from p110δ$^{Avi/Avi}$BirA$^{+/-}$ CD4$^+$ T cell blasts that had been stimulated for 2 minutes by crosslinking anti-CD3, anti-CD28 or anti-CD4 in the combinations and concentrations indicated, or that were left unstimulated or stimulated with 100 µM pervanadate. The immunoblot was probed with anti-phosphotyrosine (pTyr), anti-phosphorylated-AKT (pAKT^{T308}) and anti-AKT1. Immunoblot representative of two independent experiments.

A

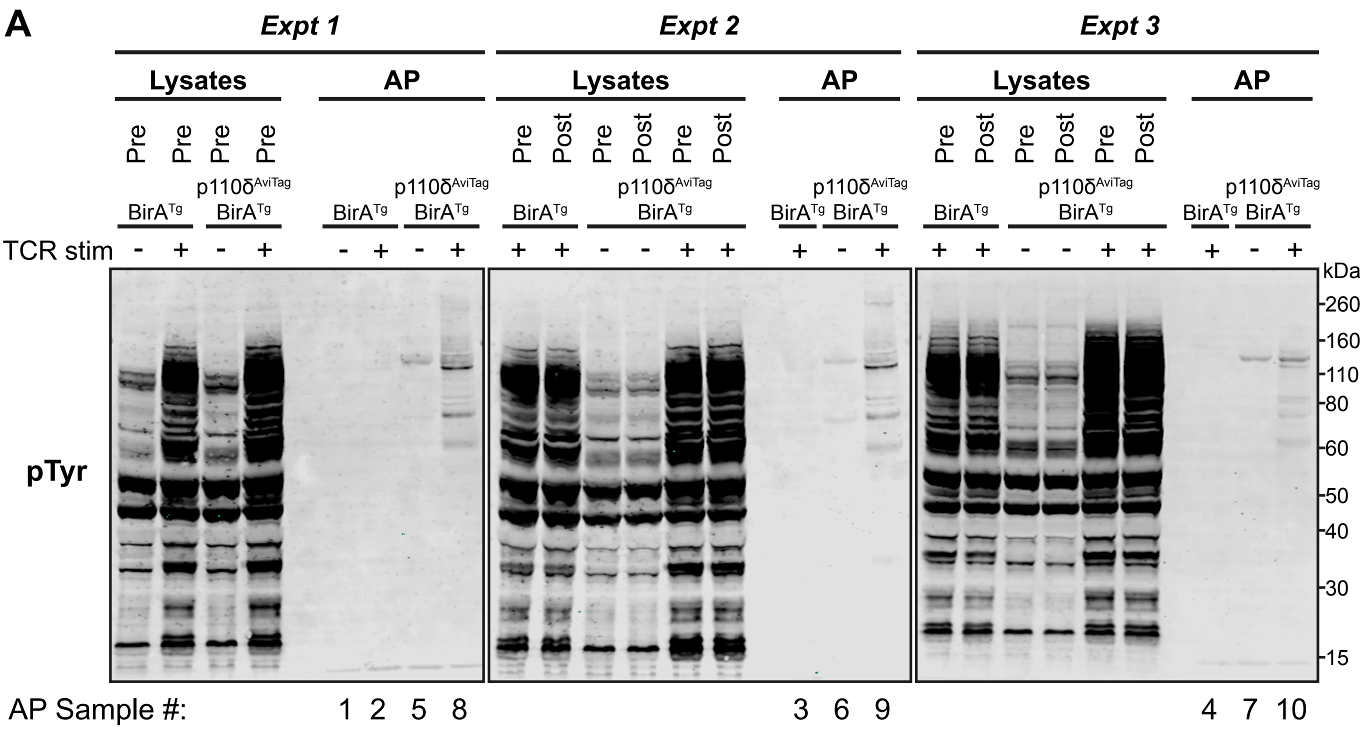

B

Expt 1: Control Unstimulated; Control Stimulated; p110δ Unstimulated; p110δ Stimulated

Expt 2: Control Stimulated; p110δ Unstimulated; p110δ Stimulated

Expt 3: Control Stimulated; p110δ Unstimulated; p110δ Stimulated

Sample	AP	Repeat #
1	Control Unstimulated	1
2	Control Stimulated	1
3	Control Stimulated	2
4	Control Stimulated	3
5*	p110δ Unstimulated	1
6	p110δ Unstimulated	2
7	p110δ Unstimulated	3
8	p110δ Stimulated	1
9	p110δ Stimulated	2
10	p110δ Stimulated	3

C

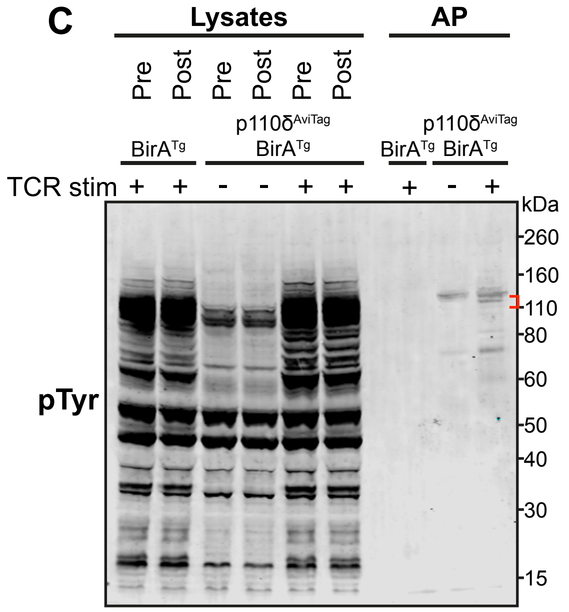

Supplementary Figure 3. Specific affinity purification of p110δ complexes from primary CD4+ T cell blasts for nLC-MS/MS analysis.

(A) Immunoblots of an aliquot of the control and p110δ affinity purifications (APs) from BirA^{Tg} and p110δ ^{AviTag}BirA^{Tg} CD4^{+} T cell blasts, respectively, that were subjected to LC-MS/MS analysis, alongside the whole cell lysate inputs (Pre) and lysates after affinity purification (Post). The cells had been stimulated for 1 minute by CD3-CD4-crosslinking (Stim; +) or were left unstimulated (-). The ten AP samples (labelled with their Sample #, corresponding to the table in (B)) were generated from three independent biological-repeat experiments (labelled Expt 1, 2 and 3). The membranes were probed with anti-phosphotyrosine (pTyr), to show consistent, robust TCR-stimulation in stimulated samples, and specific co-purification of tyrosine phosphorylated proteins in the p110 δ APs.

(B) Tables illustrating the ten AP samples (labelled with their Sample #, corresponding to the immunoblots in (A)) that were denatured, reduced, alkylated and tryptically digested to generate peptide samples. The ten peptide samples were then labelled in parallel with isobaric 10-plex TMTs before being combined for hpRP-HPLC and nLC-MS/MS analysis. *Sample 5 was labelled with a faulty TMT label and could not be included in the analysed data set.

(C) Immunoblot of an aliquot of the control and p110δ APs from BirA^{Tg} and p110δ^{AviTag}BirA^{Tg} CD4^{+} T cell blasts, respectively, that were separated by SDS-PAGE and the proteins that migrated within the gel band corresponding to 110-120 kDa were subjected to nLC-MS/MS for identification and label-free quantification. The membrane was probed with anti-phosphotyrosine (pTyr), to show consistent, robust TCR-stimulation in stimulated samples, and co-purification of tyrosine phosphorylated proteins in the p110δ APs. The red bracket represents the region of a duplicate SDS-PAGE gel that was cut out for each sample lane and subjected to nLC-MS/MS.

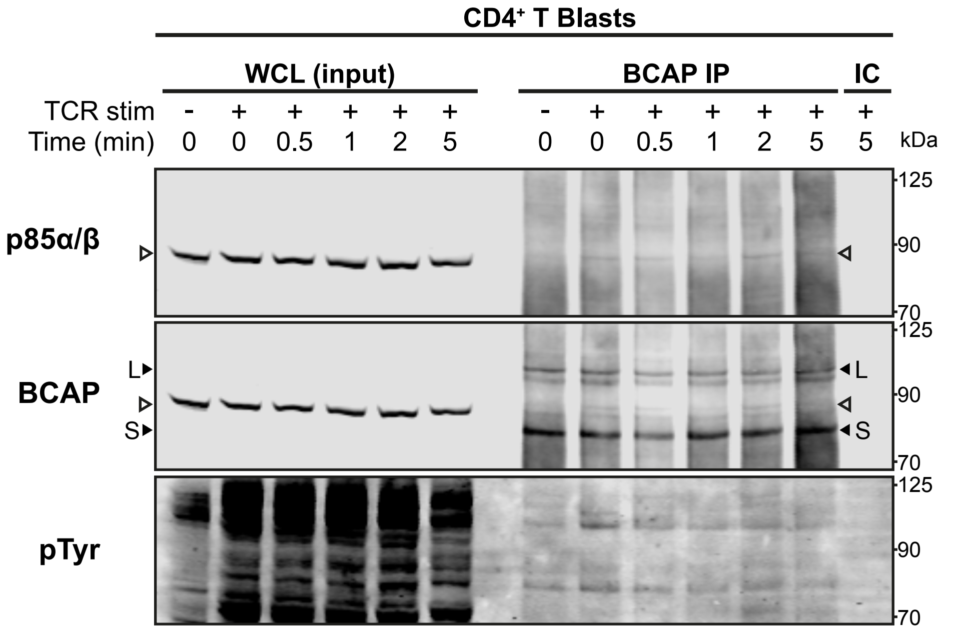

Supplementary Figure 4. Co-immunoprecipitation of p85 with BCAP from $CD4^+$ T blasts following TCR stimulation.

Immunoblot of IPs from $CD4^+$ T cell blasts using anti-BCAP (BCAP IP) or IgG isotype-control antibody (IC), alongside the whole cell lysate input (WCL input). Cells were stimulated by CD3-CD4-crosslinking (TCR stim; +) for the indicated times or left unstimulated (-). The membrane was probed consecutively with anti-pan-p85, anti-pTyr and anti-BCAP. The white arrowhead indicates p85α/β. Black filled arrowheads indicate the BCAP long (L) and short (S) isoforms. The p85α/β band is still visible after anti-BCAP blotting as the membrane was not stripped after anti-pan-p85 blotting in order to avoid protein loss from the membrane. Immunoblot from one experiment representative of 3 independent experiments.

A

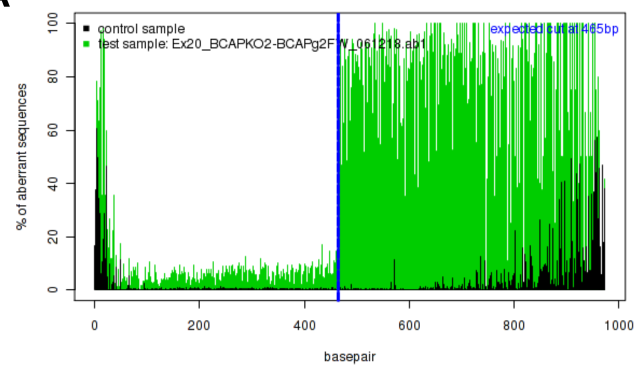

B

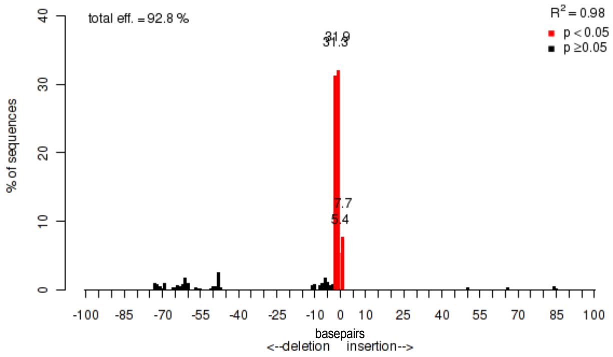

C

Biological repeat	***Pik3ap1*** **CRISPR-Cas9 KO efficiency (%)**	**Reduction in pAKT (%)**
KO 1	68.5	5.56
KO 2	92.8	28.20
KO 3	96.4	18.20
KO 4	79.1	23.93
KO 5	71.0	25.35

Supplementary Figure 5. Efficient knockout of *Pik3ap1* in activated CD4$^+$ T cells by CRISPR-Cas9-mediated gene editing.

(A-C) *Pik3ap1* DNA isolated from CRISPR-Cas9-edited (*Pik3ap1* KO) and non-edited (WT control) CD4$^+$ T cell blasts was sequenced across the ~1000bp region spanning the Cas9-cleavage site. CRISPR-Cas9 editing (KO) efficiency was determined by Tracking of Indels by Decomposition (TIDE) analysis.

(A) Representative TIDE output plot showing the proportion of *Pik3ap1* DNA sequences containing aberrant nucleotides at each position within the 1000bp region spanning the Cas9-cleavage site, in edited (*Pik3ap1* KO; green) and non-edited (WT control; black) CD4$^+$ T cell blasts. The location of the predicted Cas9-cleavage site is delineated in blue. The introduction of indels at the cleavage site following successful Cas9-cleavage will cause deviation from the wildtype sequence at each basepair beyond this position. Data is from one repeat representative of 5 biological repeats.

(B) Representative TIDE output plot showing the proportion of *Pik3ap1* DNA sequences containing insertions and deletions (indels) within the 1000bp region spanning the Cas9-cleavage site in edited (*Pik3ap1* KO) CD4$^+$ T cell blasts, versus the size of the indels. The proportion of sequences containing indels is equivalent to the knockout efficiency (%). Data are from the same *Pik3ap1* KO cells represented in (A). The R^2 value is a measure of the reliability of the values generated by TIDE analysis, and the p value indicates the statistical significance of the detection of each indel (Brinkman & Steensel 2019).

(C) Table showing the *Pik3ap1* knockout efficiency in *Pik3ap1* KO CD4$^+$ T cell blasts, generated in 5 biological-repeat experiments, alongside the percentage reduction in pAKT^{T308} detected in these cells upon TCR-stimulation compared to their corresponding non-edited control cells, as presented in Figure 4B,C.

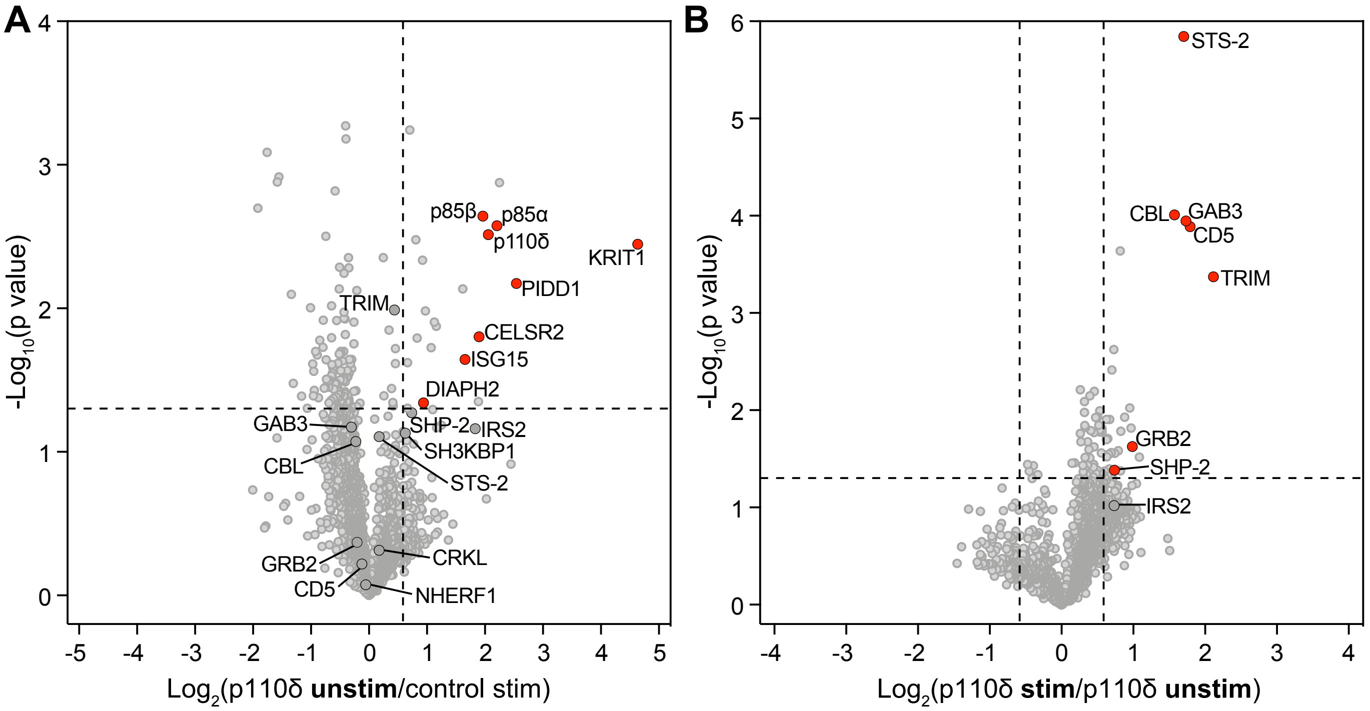

Supplementary Figure 6. The p110δ interactome in naïve CD4⁺ T cells.
(A) Volcano plot of proteins identified by mass spectrometry in APs from naïve CD4⁺ T cells. The plot shows the log_2-difference in abundance of each protein in p110δ APs from unstimulated cells compared to control APs from TCR-stimulated cells, from three independent repeat experiments, (Log_2(p110δ stim/control stim)), versus the $-Log_{10}$-p value, determined by a two-tailed Student's t-test. The thresholds used to determine specific p110 δ-interactors (upper-right quadrant) are drawn at 1.5-fold enrichment and p = 0.05. Proteins of interest are represented by red points. Proteins that had been identified as specific p110δ-interactors in TCR-stimulated naïve cells or in T cell blasts but that fell outside of the thresholds here in unstimulated naïve cells are labelled and filled in dark grey. The full t-test results can be found in *Supplementary Datasheet 3*.
(B) Volcano plot of proteins identified by mass spectrometry in p110δ APs from naïve CD4⁺ T cells. The plot shows the log_2-difference in abundance of each protein in p110δ APs from TCR-stimulated cells compared to p110 δ APs from unstimulated cells, from three independent repeat experiments, (Log_2(p110δ stim/p110δ unstim)), versus the $-Log_{10}$-p value, determined by a two-tailed Student's t-test. The thresholds used to determine TCR stimulation-induced p110δ-interactors (upper-right quadrant) are drawn at 1.5-fold enrichment and p = 0.05. The full t-test results can be found in *Supplementary Datasheet 3*.

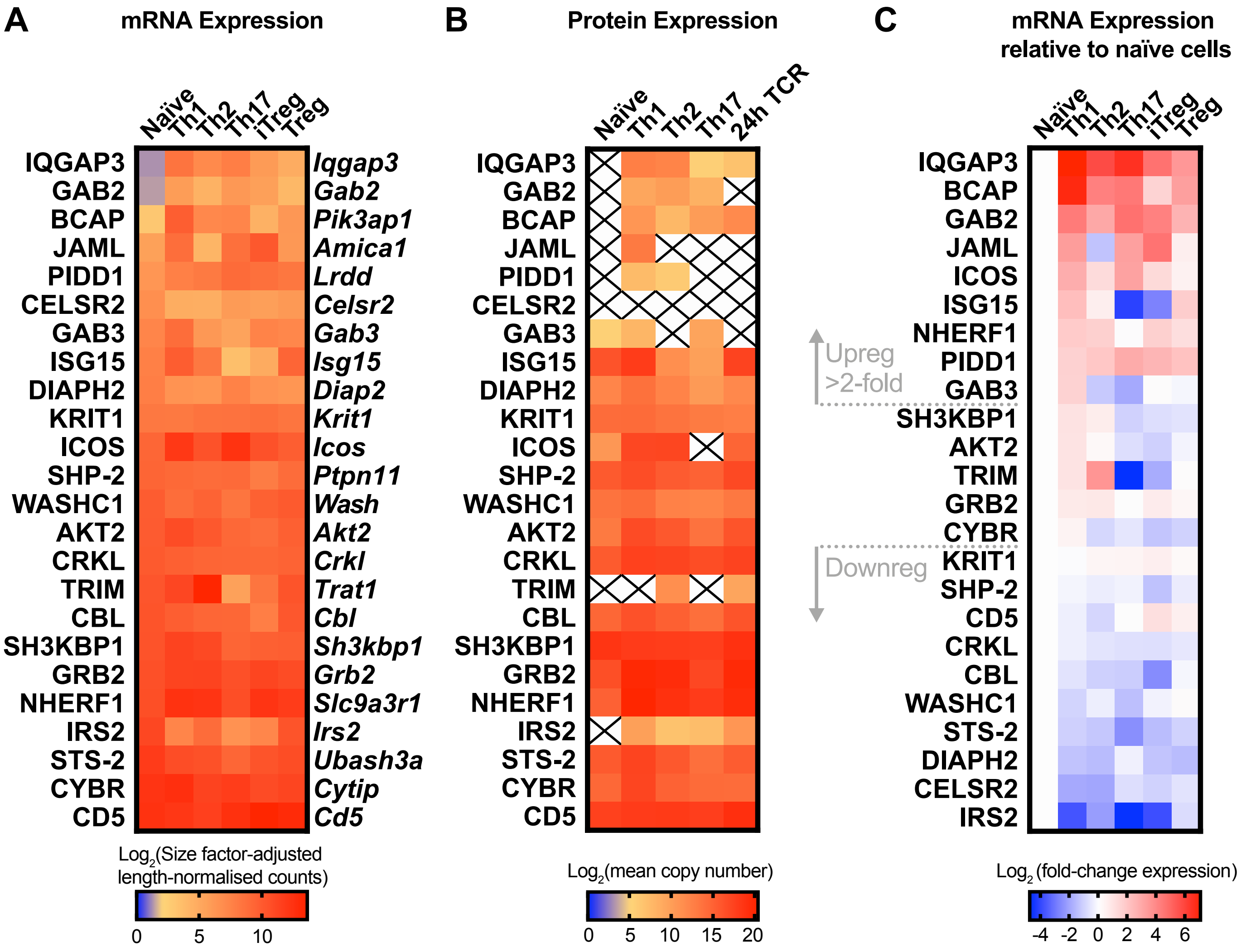

Supplementary Figure 7. Expression of p110δ-interactors in mouse CD4⁺ T cells.
(A) Heatmap visualising the $\log_2$-mRNA-expression in mouse CD4$^+$ T cells of genes encoding proteins that were identified as p110δ-interactors in naïve CD4$^+$ T cells and/or CD4$^+$ T cell blasts. Proteins are ordered by expression in naïve CD4$^+$ T cells. Data is from the publicly-available murine CD4$^+$ T cell transcriptome atlas (Th-Express; https://th-express.org; Stubbington *et al.*, 2015). Expression values in each CD4$^+$ subtype are calculated as read counts normalised by size factor and transcript length.
(B) Heatmap visualising the $\log_2$-mean protein copy number in mouse CD4$^+$ T cells of proteins that were identified as p110δ-interactors in naïve CD4$^+$ T cells and/or CD4$^+$ T cell blasts. Proteins are listed in the same order as in (A), by mRNA expression in naïve CD4$^+$ T cells. Data is taken from ImmPRes (http://immpres.co.uk; Howden *et al.*, 2019). White cells with a black cross indicate zero copy numbers were detected. 24h TCR = 24h-antigen-activated OT-II CD4$^+$ T cells.
(C) Heatmap visualising the $\log_2$-fold-change expression of genes encoding p110δ-interactors in differentiated CD4$^+$ T cell subtypes compared to naïve CD4$^+$ T cells. Proteins are ordered by their fold-change expression in Th1 CD4$^+$ T cells relative to naïve CD4$^+$ T cells. Dotted grey lines and arrows indicate genes upregulated or downregulated in Th1 cells compared to naïve cells. Data is taken from the publicly-available murine CD4$^+$ T cell transcriptome atlas (Th-Express; https://th-express.org; Stubbington *et al.*, 2015).

Supplementary Materials & Methods

nLC-MS/MS analysis of label-free samples

Peptides extracted from the 110 – 120 kDa gel slices were analysed by nano-liquid chromatography-tandem mass spectrometry (nLC-MS/MS) using the UltiMate 3000 liquid chromatography system system (ThermoFisher Scientific) interfaced via a nano-electrospray ion source onto a Q Exactive Plus hybrid Quadrupole-Orbitrap mass spectrometer (ThermoFisher Scientific). An in-house made pre-column (100 µm ID fritted fused silica packed with POROS 20 R2 reversed-phase resin) and analytical column (75 µm ID x 15 cm fused silica capillary with integrated emitter (New Objective) packed with ReproSil-Pur C18-AQ resin (2.1 µm; Dr. Maisch, Germany)) were used for peptide separation. Samples were loaded onto the pre-column in solvent A (0.1 % formic acid) at a flow rate of 6 µl/min and then separated over the analytical column at a flow rate of 250 nl/min using a linear gradient from 0 – 40 % solvent B (100 % ACN) over 30 minutes. Eluted peptides were ionised by applying a 1.8 kV voltage and introduced to the mass spectrometer as gas-phase ions. MS1 scans were only triggered when a threshold of 3e6 ions or 100 ms was reached and were acquired in the Orbitrap mass analyser with a mass resolution of 70,000 and a scan range of m/z 350-1800. The 10 most intense ions from each MS1 spectrum were isolated in the quadrupole mass analyser using a 1.2 m/z window and fragmented using higher-energy collisional dissociation (HCD) with normalised collision energy (NCE) of 27. MS2 scans of fragment ions were only triggered when a threshold of 1e5 ions or 300 ms was reached and were acquired in the Orbitrap mass analyser with a mass resolution of 17,500 and mass range starting at 100 m/z. Fragmented ions were excluded from repeated analysis for 10 seconds. MS and MS2 spectra were recorded in Xcalibur 3.0 (ThermoFisher Scientific). Raw MS data were processed in Proteome Discoverer 1.4 (ThermoFisher Scientific). Spectra were searched against the mouse UniProt database (74290 entries) and Global Proteome Machine database of common contaminants (247 entries) using Mascot. Trypsin was set as the specific protease with a maximum of three missed cleavage sites allowed. Peptide mass tolerance was set to 10 ppm and fragment mass tolerance was set to 20 mmu. Carbamidomethylation of cysteine was set as a fixed modification. Oxidation of methionine and phosphorylation of serine, threonine and tyrosine were set as variable modifications. Only high confidence peptides were used, with a 0.01 target peptide FDR, and a minimum of 1 unique peptide was required for successful protein identification. Precursor ions area detector node was used for label-free protein quantitation within Proteome Discoverer 1.4
